# TBX2 acts as a potent transcriptional silencer of tumour suppressor genes through interaction with the CoREST complex to sustain the proliferation of breast cancers

**DOI:** 10.1093/nar/gkac494

**Published:** 2022-06-10

**Authors:** Alexander J McIntyre, Charlotte Z Angel, James S Smith, Amy Templeman, Katherine Beattie, Shannon Beattie, Alice Ormrod, Eadaoin Devlin, Charles McGreevy, Chloe Bothwell, Sharon L Eddie, Niamh E Buckley, Rich Williams, Paul B Mullan

**Affiliations:** Patrick G. Johnston Centre for Cancer Research, Queen's University Belfast, Belfast BT9 7AE, UK; Patrick G. Johnston Centre for Cancer Research, Queen's University Belfast, Belfast BT9 7AE, UK; The Institute of Cancer Research, 15 Cotswold Road, Sutton, London SM2 5NG, UK; Patrick G. Johnston Centre for Cancer Research, Queen's University Belfast, Belfast BT9 7AE, UK; Patrick G. Johnston Centre for Cancer Research, Queen's University Belfast, Belfast BT9 7AE, UK; Patrick G. Johnston Centre for Cancer Research, Queen's University Belfast, Belfast BT9 7AE, UK; Patrick G. Johnston Centre for Cancer Research, Queen's University Belfast, Belfast BT9 7AE, UK; Patrick G. Johnston Centre for Cancer Research, Queen's University Belfast, Belfast BT9 7AE, UK; Patrick G. Johnston Centre for Cancer Research, Queen's University Belfast, Belfast BT9 7AE, UK; Patrick G. Johnston Centre for Cancer Research, Queen's University Belfast, Belfast BT9 7AE, UK; Patrick G. Johnston Centre for Cancer Research, Queen's University Belfast, Belfast BT9 7AE, UK; Patrick G. Johnston Centre for Cancer Research, Queen's University Belfast, Belfast BT9 7AE, UK; Patrick G. Johnston Centre for Cancer Research, Queen's University Belfast, Belfast BT9 7AE, UK; Patrick G. Johnston Centre for Cancer Research, Queen's University Belfast, Belfast BT9 7AE, UK

## Abstract

Chromosome 17q23 amplification occurs in 20% of primary breast tumours and is associated with poor outcome. The *TBX2* gene is located on 17q23 and is often over-expressed in this breast tumour subset. TBX2 is an anti-senescence gene, promoting cell growth and survival through repression of Tumour Suppressor Genes (TSGs), such as NDRG1 and CST6. Previously we found that TBX2 cooperates with the PRC2 complex to repress several TSGs, and that PRC2 inhibition restored NDRG1 expression to impede cellular proliferation. Here, we now identify CoREST proteins, LSD1 and ZNF217, as novel interactors of TBX2. Genetic or pharmacological targeting of CoREST emulated TBX2 loss, inducing NDRG1 expression and abolishing breast cancer growth *in vitro* and *in vivo*. Furthermore, we uncover that TBX2/CoREST targeting of NDRG1 is achieved by recruitment of TBX2 to the NDRG1 promoter by Sp1, the abolishment of which resulted in NDRG1 upregulation and diminished cancer cell proliferation. Through ChIP-seq we reveal that 30% of TBX2-bound promoters are shared with ZNF217 and identify novel targets repressed by TBX2/CoREST; of these targets a lncRNA, LINC00111, behaves as a negative regulator of cell proliferation. Overall, these data indicate that inhibition of CoREST proteins represents a promising therapeutic intervention for TBX2-addicted breast tumours.

## INTRODUCTION

The T-Box family of transcription factors play important roles in embryogenesis, making major contributions to the development of organs including the brain, mammary glands, thymus, liver and lungs (1). T-Box proteins can interact with target DNA via their T domain, binding the consensus half-site AGGTGTGAAA (2) and may both activate, or repress target gene transcription, depending on cellular context (3,4). T-Box 2 (TBX2) is well-known for its functions in cardiac and mammary morphogenesis, as deletion or mutation of TBX2 in these tissues results in significant congenital defects (5,6). The *TBX2* gene itself is located on chromosome 17q23, a region which exhibits extensive amplification in breast cancer and neuroblastoma, conferring poor prognosis. These amplifications result in the consistent over-expression of TBX2 and other notable oncogenes, such as *PRKCA* and *TLK2* (4,7). The TBX2 oncoprotein can promote malignant transformation by repressing the transcription of critical Tumour Suppressor Genes (TSGs) including p21^WAF1/CIP1^ and p14^ARF^, ultimately leading to senescence-bypass and immortalisation (8,9). More recently, TBX2 was found to function as a transcriptional activator in *MYCN*-amplified neuroblastoma, where it participates in a super-enhancer-driven positive feedback loop, promoting the expression of core regulatory circuitry components HAND2, ISL1, PHOX2B and GATA3 to maintain a viable cell state (10).

Although TBX2 contains a central DNA-binding T domain, our group and others have demonstrated that target gene specificity is also influenced by interaction of the protein with other transcription factors, such as Early Growth Response 1 (EGR1) and the key chromatin regulator, Retinoblastoma 1 (RB1) (11,12). In models of breast cancer, we previously found that TBX2 binds to and represses the proximal promoters of tumour suppressors N-Myc Downstream Regulated 1 (NDRG1) and Cystatin 6 (CST6), without the requirement for a T-Box element; instead TBX2 binds DNA indirectly via interaction with EGR1 at these genetic loci. This ‘exploitation’ of EGR1 function through interaction with TBX2 may be significant in the context of immortalisation, as EGR1 is also known to transcriptionally upregulate p53, p21^WAF1/CIP1^, TGF-β and PTEN; these prominent TSGs are known to perform critical roles in the induction of cellular senescence. We further demonstrated that TBX2 achieves the repression of NDRG1 by recruiting Heterochromatin Protein HP1 Gamma (HP1-γ) and components of Polycomb Repressive Complex 2 (PRC2) to the promoter region via the TBX2 C-terminal repression domain; consequently the genetic or pharmacological inhibition of PRC2 proteins resulted in transcriptional upregulation of NDRG1 and arrest of cell proliferation (13–15). As such, the indirect disruption of TBX2 function via targeting specific epigenetic modifiers presents an attractive therapeutic opportunity for the treatment of poor outcome, TBX2-overexpressing breast cancers and other tumour types with TBX2 dependency.

REST Corepressor 1 (CoREST/RCOR1) was first identified as a cofactor for RE1 Silencing Transcription Factor (REST), responsible for the transcriptional repression of neuronal genes in non-neuronal cell lineages (16). CoREST and its additional paralogs CoREST2 and CoREST3 were revealed as differential regulators of Lysine-Specific Demethylase 1 (LSD1); CoREST augments histone demethylation by LSD1, however, CoREST2 has weaker affinity for repressive cofactors Histone Deacetylases (HDACs) 1/2, while CoREST3 actively inhibits nucleosomal demethylation by LSD1 (17). Several variants of the CoREST repression complex have been isolated containing numerous histone modifiers and scaffolding proteins, including but not limited to LSD1, HDAC1, HDAC2, G9A, C-Terminal Binding Protein 1 (CtBP) and ZNF217 (18–20). The CoREST complex is highly plastic and can accomplish target promoter repression through coordinated H3K9 deacetylation, H3K4 demethylation and H3K9 methylation. Interactions are also facilitated between the CoREST and PRC2 complexes by the HOX Transcript Antisense lncRNA (HOTAIR) to coordinate CoREST activity with the repressive H3K27 methylation mark (21). Components of the CoREST complex are known to be deregulated and/or over-expressed in human breast tumours, resulting in the repression of epithelial pro-differentiation genes such as p21^WAF1/CIP1^ (22), E-cadherin (23), Grainyhead-Like 2 (GRHL2) and FOXA1 (24) to promote a malignant mesenchymal state. Unsurprisingly, the pharmacological targeting of CoREST in human cancer has become a subject of intense investigation, whereby strategies have been developed to target both individual members of the complex, or multiple cofactors simultaneously, in an attempt to recover tumour suppressor expression (25,26).

Here, we demonstrate a previously unknown mechanism of TBX2-mediated gene repression in breast tumours, whereby TBX2 physically interacts with CoREST-associated proteins LSD1, HDAC1 and the ZNF217 oncogene. We find that silencing of LSD1 and ZNF217 expression emulates the effect of TBX2 knockdown, resulting in upregulation of TBX2 targets such as NDRG1 and CST6, concomitant with diminished cell growth and clonogenic survival. Although known ‘catalytic’ inhibitors of LSD1 demethylase function exhibit no effect on growth of breast cancer models, we find that an allosteric inhibitor of the LSD1-ZNF217 interaction (SP-2509) recapitulates these phenotypes both *in vitro* and *in vivo*. Through Chromatin Immunoprecipitation sequencing (ChIP-seq) we find that while over 80% of TBX2 binding sites are concentrated at promoters, these regions show remarkably no enrichment for the T-box element; rather TBX2-bound regions are biased toward a small number of non-T-box motifs, with the most abundant being Specificity Protein 1 (Sp1), EGR1 and Nuclear Transcription Factor Y (NF-Y). Furthermore, we uncover that Sp1 is crucial for recruitment of TBX2 to the *NDRG1* promoter and subsequent repression of this gene, which is itself regulated via an internal enhancer flanked by CoREST binding sites. We also observe that ZNF217 co-occupies approximately 30% of TBX2-bound sites, a number of which contain RCOR1 and exhibit upregulation of the associated transcripts following disruption of TBX2/CoREST function. Of these transcripts we find that an uncharacterized lncRNA (LINC00111) acts as a negative regulator of cell growth and positively correlates with expression of the pro-senescence factor p21^WAF1/CIP1^. Overall these data highlight a novel therapeutic opportunity whereby poor-prognosis, TBX2-overexpressing breast tumours may be pharmacologically exploited by targeting the CoREST-dependent gene repression network, to recover normal growth control.

## MATERIALS AND METHODS

### Cell culture

Purchased cell lines were authenticated from ATCC and mycoplasma-tested prior to conducting experiments. MCF7, BT474 and MDA-MB-361 cells were cultured in Dulbecco's modified Eagle's medium (DMEM), while T47D cells were maintained in RPMI. DMEM and RPMI media were supplemented with 10% foetal calf serum, 1mM sodium pyruvate, 50 mg/ml penicillin–streptomycin and 2 mM l-glutamine (Life Technologies, Inc., Paisley, UK). MCF10A cells were grown in DMEM-F12 (1:1) supplemented with 5% Horse Serum, 100ng/ml cholera toxin, 20 ng/ml epidermal growth factor, 1 μg/ml insulin and 2.5 mM l-glutamine. MCF7 dominant-negative TBX2 cells (MCF7- DN) were grown in MCF7 media supplemented with G418, puromycin and tetracycline at 1 mg/ml each. To induce DN-TBX2 expression, cells were cultured without addition of tetracycline for indicated time periods. All were grown in 5% CO_2_ in a humidified incubator.

### Clonogenic assays, cell counts and viability assays

For clonogenic assays cells were seeded at 4000 cells/cm^2^ in six-well dishes and grown for 2 weeks (MCF7), 3 weeks (T47D) or 4 weeks (BT474 and MDA-MB-361). Cells were then fixed and stained with crystal violet and relative density quantified by converting plate scans into binary images using ImageJ (27). Cell counts were performed from 100 mm dish cultures by combining aspirated media with trypsinized cell suspension, centrifuging for 5 min at 2000 rpm and resuspending the pellets in an equal volume of 1× PBS, of which 10 μl was used for counting with a Countess™ Automated Cell Counter (Thermo Fisher Scientific). Cell viability assays were conducted by seeding in 96-well clear plastic plates at a density of 2000 cells/well (MCF7) or 3000 cells/well (T47D) for 24 h, followed by treatments for the indicated times. At the endpoint, MTT reagent (3-(4,5-dimethylthiazol-2-yl)-2,5-diphenyl tetrazolium bromide) was added to cell media at a volume ratio of 1:10 and plates incubated for 2 h at 37°C. Cell media was carefully aspirated and resulting formazan crystals resuspended using 100 μl DMSO with shaking for 30 min. Relative cell viability was then quantified by reading absorbance at 570 nm.

### esiRNA screening

Screening was conducted using a custom MISSION^®^ esiRNA panel (Sigma-Aldrich) targeting 56 known epigenetic modifying enzymes. PLK1 esiRNA was employed as a positive control for reduction of cell viability while Renilla Luciferase (RLUC) was used as a negative control. MCF7 and T47D cells were seeded at a starting density of 3000 cells/well and 4000 cells/well, respectively, in 96-well tissue culture plates (final volume: 100 μl). For transfection, 0.3 μl Lipofectamine RNAiMAX Reagent (Thermo Fisher Scientific) was added to 30 μl of OptiMEM reduced serum medium (Gibco) per well of a 96-well master plate, mixed gently and incubated at room temperature for 5 min. esiRNAs were diluted to 2 μg/ml with 6μl per well of the dilution added to the transfection reagent and OptiMEM solution, mixed gently and incubated at room temperature for 15 min. 36.3 μl of transfection mix was added to 100 μl complete medium in each well of the initial cell culture plates and the plates rocked to ensure even distribution. Cell viability was determined by MTT assay (described above) after 5–6 days incubation.

### RNA preparation and cDNA synthesis

RNA was extracted from cells using TriPure Isolation Reagent (Sigma-Aldrich) according to manufacturer's instructions. RNA was then reverse transcribed using the Transcriptor First Stand cDNA synthesis kit (Roche, UK) following the manufacturer's instructions.

### Real-time quantitative PCR

Relative quantitative PCR was performed on a 96-well plate (MJ Research, Waltham, MA, USA) on the LightCycler 96 System (Roche Life Science), and analysed using Roche Lightcycler 96 software according to the manufacturer's instructions. Gene expression was normalized relative to expression changes in SDHA (Succinate Dehydrogenase Complex Flavoprotein Subunit A). Primer sequences are shown in Supplementary Table S1.

### Western blot analysis and antibodies

To extract protein, cell pellets were lysed with RIPA (5 mM EDTA pH 8.0, 1% IGEPAL CA-630, 150 mM NaCl, 0.5% Na deoxycholate, 0.1% SDS, 50 mM Tris–HCl pH 8.0) supplemented with cOmplete Mini EDTA-free Protease Inhibitor Cocktail (Roche Life Science) for 15 min on ice, followed by centrifugation for 15 min at 13 000 rpm and 4°C. Supernatant was transferred to a new 1.5 ml Eppendorf and protein quantified using Bradford Reagent (Bio-Rad). Lysates were denatured with the addition of 2× LDS Sample Buffer (Thermo Fisher Scientific) and heating for 5 min at 95°C. 30 μg of lysate was resolved by PAGE using the Bolt Bis–Tris system (Thermo Fisher Scientific) following the manufacturer's instructions. Proteins were transferred to Protran nitrocellulose membranes (Amersham) using a Mini-PROTEAN cell (Bio-Rad) following the manufacturer's instructions. Membranes were blocked for 1 h in 1× TBS with 0.02% Tween-20 (TBS-T) and 5% Marvel prior to overnight incubation at 4°C with primary antibody diluted according to manufacturer's instructions. Membranes were washed three times (10 min each) in TBS-T, prior to incubation with species-matched Anti-IgG HRP-linked secondary antibody (Cell Signaling Technology) diluted 1:2000 in TBS-T with 5% Marvel for 1 h at room temperature. Membranes were washed as before and developed with the addition of Immobilon Crescendo Western HRP substrate (Millipore) using the G:BOX Chemi system (Syngene) following the manufacturer's instructions. The primary antibodies: LSD1 (C69G12), NDRG1 (#5196), EZH2 (AC22) and HP1-γ (#2619) were obtained from Cell Signaling Technology; HDAC1 (10E2), p53 (DO-1), p21^WAF1/CIP1^ (C-19) and TBX2 (D-3) were obtained from Santa Cruz Biotechnology; CDK1 (#610038) was obtained from BD Biosciences; H3K4me2 (Y-47) and Sp1 (EPR22648-50) were obtained from Abcam; FLAG-M2 (#F1804) and GRHL2 (HPA004820) were obtained from Sigma-Aldrich; ZNF217 (#720352) was obtained from Thermo Fisher Scientific; KAP1 (BL553) was obtained from Bethyl Laboratories.

### Co-immunoprecipitations

Cells were seeded on 140mm dishes prior to harvesting. Cells were harvested at 4°C by scraping into 15ml falcon tubes and centrifugation at 1000 rpm for 5 min at 4°C. Pellets were washed with 5 ml cold 1× PBS and centrifuged as before. The washed pellets were resuspended in 1ml egg lysis buffer (ELB) (300 mM NaCl, 0.1% IGEPAL CA-630, 50 mM HEPES, 1 mM EDTA, 0.5 mM DTT) supplemented with cOmplete Mini Protease Inhibitor Cocktail + PhosSTOP (Roche Life Science) and transferred to 1.5 ml Eppendorf tubes. Following 15 min of incubation on ice, lysates were centrifuged at 13 000 rpm for 15 min at 4°C and the resulting supernatants transferred to new 1.5 ml Eppendorf tubes for protein quantification. Per IP, 1mg of lysate was added to a fresh 1.5 ml Eppendorf tube and volume-adjusted to 500 μl with ELB supplemented with protease inhibitor. 30 μl Dynabeads Protein G (Thermo Fisher Scientific) were prepared by washing three times in 1 ml ELB and then added to the 1 mg of lysate, followed by rotation for 4 h at 4°C to pre-clear the lysate. Beads were then removed magnetically and the pre-cleared lysate transferred to a new 1.5 ml tube. Lysate was incubated with 2 μg target antibody or species-matched control IgG by rotating at 4°C overnight. 30 μl Dynabeads Protein G were then prepared as before, added to the lysate tube and rotated for 4 h at 4°C to capture antibody-protein complexes. The beads were washed five times with ELB at 4°C and all liquid removed by magnetic isolation. Bead-antibody-protein complexes were denatured by addition of 20 μl 2× LDS Sample Buffer (Thermo Fisher Scientific) and 20 μl ELB with heating for 10 min at 95°C. Western blotting was conducted as described comparing 10 μl of each IP against 30 μg of input lysate.

### siRNA knockdowns

Cells were transfected using Lipofectamine RNAiMAX Reagent (Thermo Fisher Scientific) according to manufacturer's instructions. Protein and/or RNA was collected from cells following treatment at indicated time points with 25 nM siRNA. siRNA sequences are listed in Supplementary Table S1.

### MCF7 xenograft experiments

All experiments were performed under an ASPA-approved protocol. Female SCID mice were each implanted subcutaneously with a 2 mg estradiol (E2) pellet one week prior to subcutaneous implantation of two million MCF7 cells into the lower flank. Once tumours reached ∼100 mm^3^, groups of 10 mice were treated daily with vehicle (45% PEG-400, 10% EtOH, 45% PBS pH 9.0) or 40 mg/kg of SP-2509 by intraperitoneal injection from Monday to Friday for 4 weeks. Tumour measurements and body weight measurements were recorded twice a week, and all animals were sacrificed at the end of week 4.

### Immunohistochemistry (IHC)

Tumour samples were blinded prior to staining and quantification to prevent unconscious bias. Staining was carried out on whole face tissue sections, cut from formalin fixed paraffin-embedded blocks. Tissue sections were cut at 5 μm using a LEICA RM21215 RTS microtome and dried overnight at 40°C. Tissue slides were rehydrated from xylene through a series of alcohols (100–50%) to water. IHC was performed using antigen retrieval under pressure-cooking in 0.1 M sodium citrate pH 6.0 and 3% hydrogen peroxide. Slides were blocked in avidin-biotin and serum prior to overnight incubation with Ki-67 antibody (ab15580, abcam, 1:150 dilution). Slides were then incubated with biotinylated secondary antibody (Vector Labs) at a dilution of 1:200. ABC reagent was added and DAB staining carried out under a microscope. All IHC slides were then counterstained with haematoxylin prior to dehydration and mounting. Ki-67 images were taken at 20× magnification and stitched to a single image using LASV-LEICA 4.8 UK software. Stitched images were quantified using QuPath software to evaluate the amount of overall positive Ki-67 nuclear staining against negative staining.

### Chromatin immunoprecipitation (ChIP)

Per IP, 20 million cells were harvested by trypsinisation and centrifuged at 1200 rpm in a 15ml falcon. The cells were washed twice in PBS and resuspended in PBS at a final volume of 9.4 ml. 625 μl fresh 16% methanol-free formaldehyde (Thermo Fisher Scientific) was added to the cell suspension which was then rotated for 10 min at room temperature to fix. The formaldehyde was quenched by adding 4 ml ice-cold 2.5 M glycine and rotating at room temperature for 5 min. Fixed cells were collected by centrifugation at 1600 rpm for 5 min at 4°C and washed twice with 10 ml ice-cold PBS. The cell pellet was then resuspended in 10 ml ice cold cytoplasmic lysis buffer (50 mM HEPES–KOH pH 7.5, 150 mM NaCl, 1 mM EDTA, 1% Triton X-100, 0.1% Na deoxycholate, 0.1% SDS) and rotated at 4°C for 15 min. Cells were pelleted at 2300 rpm for 10 min, the lysis buffer removed and cytoplasmic lysis repeated a second time with fresh lysis buffer. The pellet was resuspended in 10 ml ice cold nuclear lysis buffer (50 mM HEPES–KOH pH 7.5, 150 mM NaCl, 1 mM EDTA, 1% Triton X-100, 0.1% Na deoxycholate, 1% SDS) and rotated at 4°C for 15 min. Cells were pelleted at 2300 rpm for 10 min, the lysis buffer removed and the chromatin pellet resuspended in 1.8 ml fresh nuclear lysis buffer. The chromatin suspension was divided into 1.5 ml Bioruptor Plus TPX microtubes (Diagenode) at 300 μl per tube and loaded into a Bioruptor UCD-500 (Diagenode) pre-chilled to 4°C. Chromatin was sonicated for 33 cycles of 30 s on/30 s off, Triton X-100 added to each TPX tube to a final concentration of 1% and the tubes centrifuged at 13 000 rpm for 10 min to sequester SDS. Chromatin was then pooled to a single 2 ml Eppendorf DNA LoBind tube. 30 μl Dynabeads Protein G were prepared by washing twice in 1ml beads wash buffer (PBS, 0.1% Triton X-100), added to chromatin and the mixture rotated for 2 h at 4°C to pre-clear the chromatin. Beads were removed on a magnet and the chromatin transferred to a new 2 ml LoBind tube, with 30 μl retained as an input sample. 6 μg target ChIP antibody or 6 μg species-matched IgG control was added to the chromatin and the tube rotated overnight at 4°C. 60 μl Dynabeads Protein G were then prepared by washing twice in 1 ml beads wash buffer, added to chromatin and the mixture rotated for 2–4 h at 4°C to capture antibody-DNA complexes. The beads were washed three times in 1 ml cytoplasmic lysis buffer, twice in 1 ml high salt buffer (50 mM HEPES–KOH pH 7.5, 350 mM NaCl, 1 mM EDTA, 1% Triton X-100, 0.1% Na deoxycholate, 0.1% SDS), once in lithium chloride buffer (10 mM Tris–HCl pH 8.0, 250 mM LiCl, 1 mM EDTA, 0.5% IGEPAL CA-630, 0.5% Na deoxycholate) and once in TE buffer (10 mM Tris–HCl pH 8.0, 0.1 mM EDTA). To purify ChIP DNA, beads and input sample were suspended in elution buffer (50 mM Tris–HCl pH 7.5, 10 mM EDTA, 1% SDS) to a final volume of 300 μl and incubated with shaking at 1000 rpm at 65°C overnight. 50 μl of 20 mg/ml Proteinase K Solution (Thermo Fisher Scientific) was added to each of the tubes, which were then incubated for 2 h shaking as before at 55°C. 300 μl phenol chloroform isoamyl alcohol (25:24:1, v/v) was mixed with each sample, incubated for 3 min at room temperature and the tubes centrifuged at 13 000 rpm for 5 min at room temperature. 280 μl of aqueous phase containing DNA was added to 300 ul ice-cold isopropanol, 30 μl 3 M sodium acetate and 1 μl of GlycoBlue Coprecipitant (Thermo Fisher Scientific). Samples were briefly vortexed, frozen at –80°C for 30 min and centrifuged at 13 000 rpm at 4°C for 30 min to pellet DNA. Purified DNA was washed gently in 1 ml 100% ethanol, air-dried and finally resuspended in 50 μl TE buffer. The ChIP antibodies: TBX2 (ab33298) was obtained from Abcam; Normal Rabbit IgG (#2729) was obtained from Cell Signaling Technology. Two independent biological replicates of ChIP material were obtained prior to RT-qPCR or next generation sequencing.

### ChIP-seq

1ng of input and ChIP DNA were taken to generate Illumina-compatible indexed libraries using the MicroPlex Library Preparation Kit v2 (Diagenode) following the manufacturer's instructions. Libraries were size-selected to an average fragment size of 300-400 bp using KAPA Pure Beads (Roche) which was confirmed with an Agilent High Sensitivity DNA Kit (Agilent Technologies) following the manufacturer's instructions. The molarity of cluster-competent library molecules was calculated using the NEBNext Library Quant Kit for Illumina (New England BioLabs Inc.) and the individual libraries pooled for multiplex sequencing at an equimolar ratio to 4 nM final concentration. The ChIP library pool was sequenced with an Illumina Next Seq 500 High Output Kit for 75 cycles at ∼40 million single-end reads per sample/index. Sequencing FASTQ files were de-multiplexed on Sanger indexes and the quality of sequencing confirmed with the FastQC tool (28) prior to downstream analysis.

### Bioinformatic analysis of ChIP-seq data

Single-end FASTQ files obtained from in-house MCF7 ChIP-seq of Input vs TBX2 (2 biological replicates each) were aligned to human genome hg38 primary assembly using Bowtie2 (29) (end-to-end mode). FASTQ files for MCF7 ZNF217 and RCOR1 ChIP-seq with matched control samples (two biological replicates each) were obtained from ENCODE under accession numbers ENCBS764AUT, ENCBS773JGQ, ENCBS295PFW, ENCBS295PFW, ENCBS216AOQ, ENCBS034XKZ, ENCBS747ZRJ and ENCBS609QTY. TBX2 ChIP-seq data from Kelly cells were obtained from GEO under accession numbers GSM2486165 and GSM2915911. Public FASTQ files were aligned to hg38 using Bowtie2 as before. All BAM files were filtered on a minimum MAPQ of 10 to retain unique alignments with no more than 3 mismatches. NarrowPeaks were called on BAM files of individual ChIP replicates vs matched input controls using MACS2 (30) callpeak (tag extension size = 250 bp, keep-dup = auto). Peaks were then called on pooled replicates in MACS2 callpeak using previous settings, outputting additional bedGraph profiles normalized to counts per million (CPM). bedGraph files were converted to bigWig for genome browser viewing of ChIP-enriched regions. Reproducible peaks in pooled data were classed as regions overlapping at least 1bp with narrowPeaks called in each individual replicate. Peak summits were profiled with ChIPseeker (31) using Ensembl GTF annotation (obtained from UCSC) to determine the fraction of peaks falling into intergenic/intragenic/promoter regions. Genes associated with promoter regions were analysed using Enrichr (32) to determine significant signalling pathway/network terms against multiple databases. For *de novo* motif discovery, peak summits were extended 100bp in each direction to create 200 bp windows and associated nucleotide sequences extracted in FASTA format. FASTA sequences were processed with RepeatMasker (33) to hide interfering elements prior to analysis in MEME suite (http://meme-suite.org/). FASTA sequences were analysed with the STREME tool to find enriched motifs 8–15 bp in length with *P*-value <0.05, and matches to known motifs scored against the HOCOMOCO Human v11 CORE database. Discovered motif PWMs were re-analysed in CentriMo by scoring against 500 bp FASTA sequences centred on peak summits; motifs were classed as centrally enriched if sequence matches were concentrated in the 200 bp region surrounding the summit, relative to the rest of the peak width. Genomic coordinates of the CentriMo output were then used to identify individual peak locations for each motif. To enhance visualisation of the *NDRG1* locus, MCF7 tracks for POLR2A ChIA-PET, H3K27Ac and H3K4me1 were downloaded from ENCODE under accession numbers ENCFF877DPA, ENCFF063VLJ and ENCFF328PXQ, respectively.

### Statistics

Statistically analysed experiments were performed with at least three biological replicates unless otherwise stated. Statistical analyses were performed by two-tailed Student's *t*-test. Values with *P* <0.05 or smaller are considered as statistically significant.

## RESULTS

### LSD1 interacts with TBX2 and is required for breast cancer cell survival

**Figure 1.**
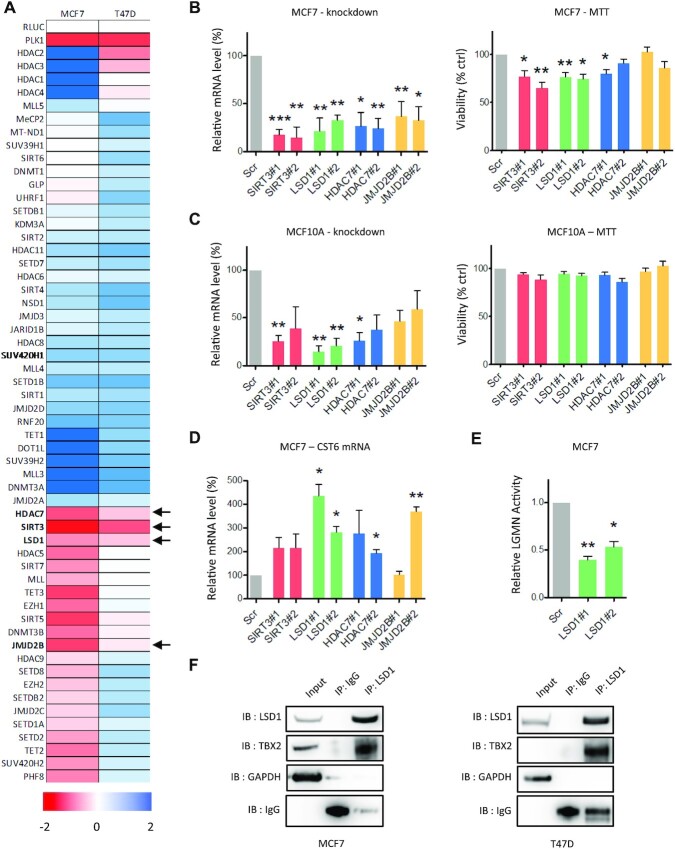
LSD1 interacts with TBX2. (**A**) MTT cell viability screen of TBX2-dependent MCF7 and T47D cells transfected with MISSION® esiRNA panel targeting 56 epigenetic modifiers. Heatmap values are normalized to RLUC negative control esiRNA and displayed as log_2_ fold-change, contiguous with colour scale below. PLK1 esiRNA was used as negative control for reduced viability. Arrows indicate growth dependencies taken forward for validation. (**B**) RT-qPCR validation of SIRT3/LSD1/HDAC7/JMJD2B knockdown in MCF7 at 48 h. Each column represents mRNA expression level relative to scrambled control (Scr) using primers corresponding to below siRNA target (representative Scr control shown for scale). Time-matched MTT cell viability measurements are shown to the right for each siRNA as normalized to Scr control. (**C**) RT-qPCR validation of knockdowns and matched MTT cell viability at 48h in non-malignant MCF10A cells, as described above for MCF7. (**D**) RT-qPCR mRNA measurement for TBX2 target CST6 at 48h following SIRT3/LSD1/HDAC7/JMJD2B knockdown relative to Scr control in MCF7. (**E**) Effect of LSD1 knockdown on activity of Legumain (LGMN), the protease activity of which is directly inhibited by CST6. MCF7 cells were treated for 120h with Scr or LSD1 siRNAs, after which lysates were subjected to LGMN activity assay by addition of fluorogenic LGMN substrate (Z-Ala-Ala-Asn-AMC). LGMN activity is shown relative to Scr control. (**F**) Western blot of Co-IP assay in MCF7 and T47D cells. LSD1 was precipitated from lysates using anti-LSD1 antibody, with species-matched IgG as control IP. Samples were immunoblotted for LSD1 and TBX2, with GAPDH serving as negative control. Error bars represent mean ± s.e.m. of three independent experiments. *P< 0.05; **P< 0.01; unmarked = not significant.

Transcription factors have long been recognized as challenging drug targets owing to their inaccessible cellular localisation, their large protein-protein interfaces and lack of intrinsic enzymatic activity (34). We previously set out to address this matter by characterizing the mechanisms through which the TBX2 oncogene represses transcription in breast tumours; this uncovered the TBX2-dependent recruitment of a PRC2-like complex to the NDRG1 promoter, containing enzymes such as Enhancer of Zeste Homolog 2 (EZH2) and G9A/GLP, both of which can be targeted pharmacologically (15). We therefore wanted to ascertain whether TBX2 could form other repression complexes, which may represent additional therapeutic strategies. Initially a viability screen was performed in TBX2-dependent MCF7 and T47D breast cancer models, employing an endoribonuclease siRNA (esiRNA) library of 56 known epigenetic modifiers; any resulting ‘hits’ whose knockdowns reduced cell viability could represent potential functional interactors of TBX2 (Figure 1A). From this screen four hits, required for viability of both cell lines were taken forward for downstream validation; HDAC7, Sirtuin-3 (SIRT3), LSD1 and Jumonji Domain-Containing 2B (JMJD2B)/KDM4B. Viability assays performed 48 h after knockdown of each candidate with two independent siRNAs found a partial requirement of SIRT3 and LSD1 for growth of MCF7 cells, but not in MCF10A normal breast cells (Figure 1B and C). However, from these short term knockdowns, LSD1 knockdowns exhibited the most significant and reproducible effect on mRNA upregulation of the TBX2 target CST6 (13), coincident with reduction in activity of the enzyme, Legumain, normally inhibited by CST6 (Figure 1D and E, respectively). This suggested that LSD1 could be a possible interacting partner of TBX2. Indeed, this hypothesis was confirmed by co-immunoprecipitation assays in MCF7 and T47D models, which demonstrated a physical interaction between TBX2 and LSD1 (Figure 1F). Together these data suggested that TBX2 may interact with multiple epigenetic regulators and that LSD1 inhibition could represent a viable strategy to target TBX2 dependent breast cancers.

**Figure 2.**
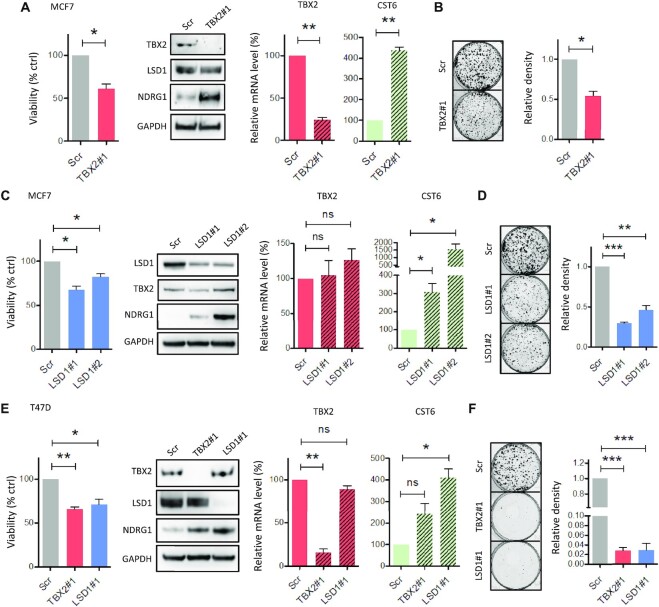
LSD1 knockdown phenocopies loss of TBX2. (**A**) MCF7 cells were treated with TBX2 siRNA or scrambled control for 72 h. Relative cell viability was measured by MTT assay; effects on protein level of TBX2, LSD1 and TBX2 target NDRG1 were detected by western blot; effects on mRNA of TBX2 and CST6 were assessed by RT-qPCR. (**B**) MCF7 Clonogenic assay following treatment with TBX2 siRNA for 2 weeks, with effect on colony density shown in representative images and adjacent bar chart. (**C**) MCF7 cells were treated with 2 independent LSD1 siRNAs or scrambled control for 72 h. Relative cell viability, protein levels of TBX2/LSD1/NDRG1 and mRNA levels of TBX2/CST6 were assessed as described above. (**D**) MCF7 Clonogenic assay following treatment with LSD1 siRNAs for 2 weeks, with effects on colony density shown as above. (**E**) T47D cells were treated with TBX2 siRNA, LSD1 siRNA or scrambled control for 120h. Relative cell viability, protein levels of TBX2/LSD1/NDRG1 and mRNA levels of TBX2/CST6 were assessed as described above. (**F**) T47D Clonogenic assay following treatment with TBX2 or LSD1 siRNA for 3 weeks, with effects on colony density shown as above. Error bars represent mean ± s.e.m. of three independent experiments. *P< 0.05; **P< 0.01; ***P< 0.001; ns = not significant. GAPDH serves as loading control for all western blots.

Interactions between transcription factors and other chromatin-regulatory proteins often influence protein stability in transcriptional complexes (35,36). We therefore determined whether this was the case for the TBX2-LSD1 interaction. Firstly, MCF7 cells treated with siRNA targeting TBX2 for 72h displayed reduced proliferation, concomitant with upregulation of target genes NDRG1 and CST6; however, western blot analysis showed this occurred independently of effects on LSD1 protein level (Figure 2A). Likewise, knockdown of LSD1 with two independent siRNAs phenocopied the effects of TBX2 depletion but with no associated reduction of TBX2 RNA or protein (Figure 2C). Clonogenic assays also confirmed that LSD1 and TBX2 knockdown had a comparable impact on long-term survival with a significant reduction of colony numbers (Figure 2B and D). These phenomena were consistent in a second cell line (T47D) wherein knockdown of TBX2 or LSD1 resulted in a similar reduction of cell proliferation and upregulation of NDRG1/CST6, without impacting on each other's stability at the protein level (Figure 2E). Crystal violet growth assays again confirmed that TBX2 and LSD1 were essential for clonogenic survival in T47D cells (Figure 2F). Taken together these data indicated that although TBX2 and LSD1 did not have a mutual requirement for protein stability, the interaction between the two factors may be important for repression of TBX2 target genes and maintenance of breast cancer cell growth.

### Allosteric LSD1 inhibitor SP-2509 de-represses TBX2 targets and inhibits estrogen-dependent breast tumour growth *in vivo*

**Figure 3.**
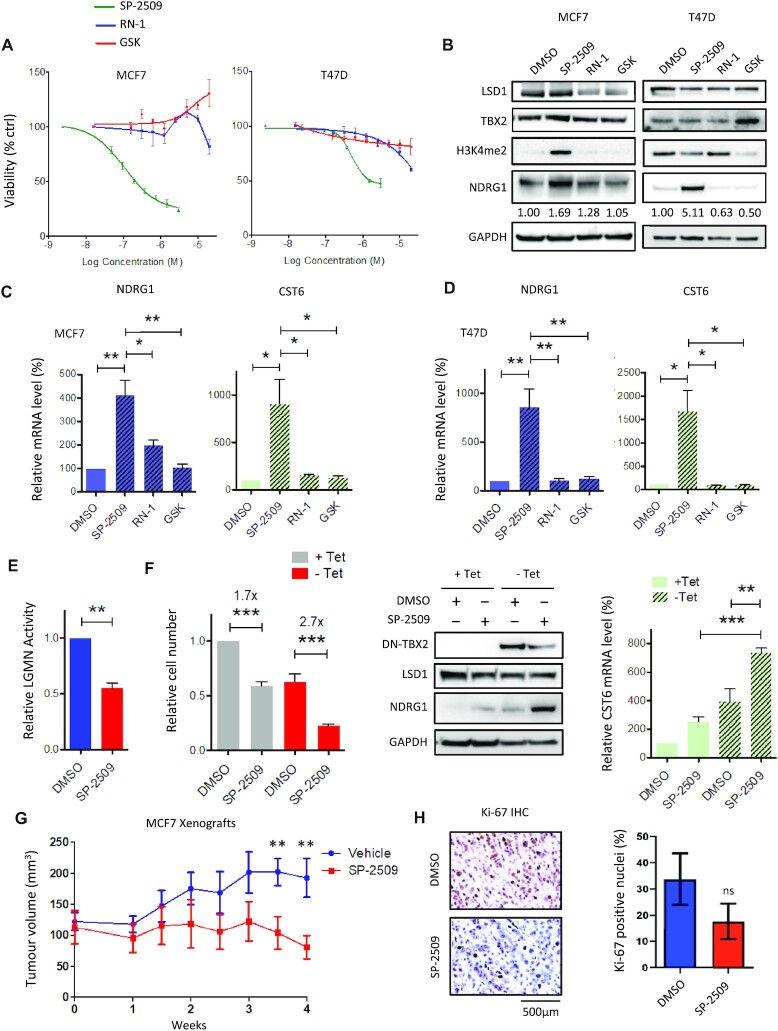
An allosteric LSD1 inhibitor enhances expression of TBX2-repressed genes and prevents breast tumour growth *in vivo*. (**A**) MTT viability assay of MCF7 and T47D cells treated with increasing doses of allosteric (SP-2509) or catalytic (RN-1/GSK) inhibitors of LSD1 for 72h, with values shown relative to no-drug control (DMSO). (**B**) Western blot from MCF7 and T47D lysates, comparing effects of SP-2509 IC50 (≤1μM) with 20μM RN-1/GSK on protein expression of LSD1, TBX2, H3K4me2 and NDRG1 at 72 h versus DMSO control. Numbers below NDRG1 lane correspond to fold change in NDRG1 densitometry relative to GAPDH. (**C**) RT-qPCR of MCF7 cDNA comparing effects of SP-2509 IC50 (250nM) with 20 μM RN-1/GSK on mRNA expression of NDRG1 and CST6 at 72 h vers DMSO control. (**D**) RT;qPCR of T47D cDNA comparing effects of SP-2509 IC50 (1 μM) with 20 μM RN-1/GSK as described above. (**E**) LGMN activity assay following treatment with 250 nM SP-2509 for 120 h relative to DMSO control in MCF7. (**F**) MCF7-DN cells were induced to express DN-TBX2 (–Tet) or not induced (+Tet) for 48 h prior to treatment with DMSO or 500 nM SP-2509 for a further 72 h. Bar graph illustrates effects on cell number relative to uninduced DMSO control. Mean fold-change in cell number with SP-2509 relative to DMSO control is shown for the +Tet and –Tet groups. Matched western blot demonstrates effects of treatments on protein levels of FLAG-DN-TBX2, LSD1 and NDRG1; matched RT-qPCR demonstrates effects of treatments on mRNA level of CST6. Error bars represent mean ± s.e.m. of three independent experiments. (**G**) Mean tumour volume of MCF7 xenografts treated with vehicle control or 40mg/kg SP-2509 over a 4-week period. Mice were sacrificed at the end of week 4 and tumour tissues subjected to immunohistochemical staining for proliferation marker Ki-67. (**H**) Representative images shown for each treatment group; percentage of Ki-67 positive nuclei were quantified and are displayed in adjacent bar graph. Error bars represent mean ± s.e.m. of ten individual mice. *P< 0.05; **P< 0.01; ***P< 0.001; ns = not significant. GAPDH serves as loading control for all western blots.

Having identified LSD1 as a novel interactor of TBX2 required for cell proliferation, we addressed whether this dependency could be targeted pharmacologically by assaying the LSD1 inhibitors SP-2509, RN-1 and GSK-LSD1. RN-1 is a derivative of tranylcypromine which forms covalent adducts with the cofactor Flavin Adenine Dinucleotide (FAD), inhibiting the amine oxidase function of LSD1 responsible for H3K4 demethylation (37). GSK-LSD1 is also tranylcypromine-based and is classified as a catalytic inhibitor of LSD1; however, recent work finds its anti-cancer effect is primarily due to allosteric disruption of the LSD1 interaction with AML oncoprotein Growth Factor Independent 1B (GFI1B) (38). In contrast, SP-2509 is derived from benzohydrazide and interacts allosterically with the histone H3-binding pocket of LSD1; the anti-tumour activity of the drug was lately attributed to inhibition of the interaction between LSD1 and ZNF217 (39). To begin with, MCF7 and T47D cells were exposed to increasing concentrations of LSD1 inhibitors (Figure 3A). Of the three inhibitors tested, only SP-2509 displayed efficacy in reducing cell proliferation with an IC50 of ∼250 nM in MCF7 and 1μM in T47D at 72 h. Both cell lines were highly resistant to the effects of RN-1 and GSK-LSD1, as IC50 could not be reached at the maximum 20 μM dose; indeed, GSK-LSD1 actually enhanced the growth of MCF7 (likely due to off-target effects). Western blot analysis revealed that treatment with IC50 doses of SP-2509 resulted in marked upregulation of NDRG1, however, no such effect was present with 20 μM RN-1 or 20 μM GSK-LSD1. The protein level of TBX2 remained unaffected by disruption of LSD1 activity by SP-2509, analogous to our previous observations concerning TBX2 expression following LSD1 knockdown. While NDRG1 upregulation correlated with global increase in H3K4 dimethylation (H3K4me2) in MCF7 cells, it did not correlate with this histone mark in T47D cells, implying that global changes in H3K4me2 occurred as an indirect effect of SP-2509 treatment (Figure 3B). Regardless of H3K4me2 levels, the treatment of both cell lines with SP-2509 IC50 led to significant transcriptional upregulation of targets NDRG1 and CST6, while little to no effect was seen with RN-1 or GSK-LSD1 at 20μM (Figure 3C and D). Accordingly, SP-2509 treatment resulted in significantly reduced activity of the CST6 target Legumain (Figure 3E). We next addressed whether SP-2509 could exert TBX2-dependent phenotypes with the use of a Tet-off inducible model; this expresses a dominant-negative TBX2 protein (DN-TBX2) comprised of amino acids 1–301 including the intact T domain, but lacks amino acids 302–701 wherein the repression domain is located (3). Interestingly, the antiproliferative effect of SP-2509 was more pronounced in MCF7 cells which were induced to express truncated TBX2 (Figure 3F), alongside upregulation of targets NDRG1 and CST6 to a significantly higher level than the individual treatments (Figure 3F). Notably DN-TBX2 had a negligible impact on LSD1 protein expression as previously observed with TBX2 knockdown (Figures 2C and 3F), indicating this enhanced sensitivity to SP-2509 was not due to downregulation of the drug target. Given that SP-2509 achieved cell growth arrest and upregulation of TBX2 targets at relatively low concentrations, it was deemed a suitable candidate for *in vivo* experimentation. To determine whether SP-2509 could inhibit breast tumour growth *in vivo*, Estrogen-supplemented mice were xenografted with MCF7 cells and treated with 40 mg/kg SP-2509 or vehicle for four weeks (Figure 3G). Indeed at 3–4 weeks treatment with the LSD1 inhibitor, we observed a significant reduction in average tumour volume versus vehicle treated mice; importantly, we did not observe toxicity-associated changes in body or organ weight (Supplementary Figure S1). Subsequently, endpoint immunohistochemical staining found that expression of proliferation marker Ki-67 in SP-2509-treated tumour tissue was approximately half the level observed in the vehicle control group (Figure 3H). Overall these *in vitro* and *in vivo* data provided evidence that specific targeting of LSD1, at the H3 allosteric site via SP-2509, can lead to effective upregulation of TSGs and may be a viable therapeutic option for exploiting TBX2 dependency in breast cancers.

### TBX2 interacts with components of the CoREST complex

**Figure 4.**
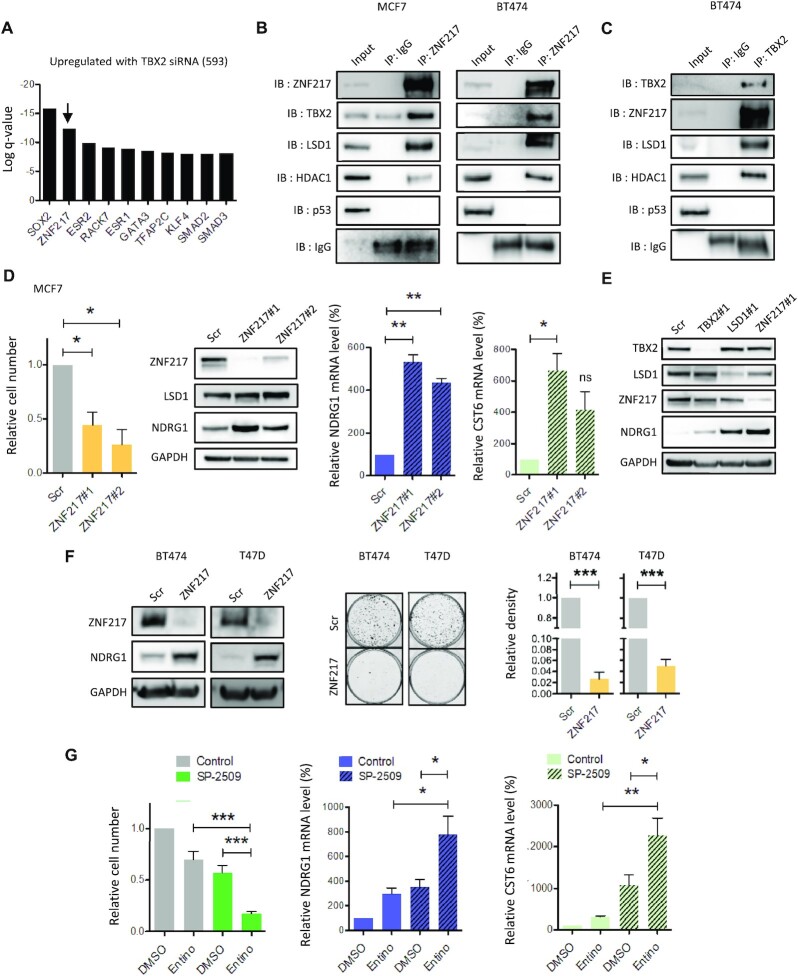
TBX2 interacts with components of the CoREST repression complex. (**A**) TF signature enrichment (ChEA database) of genes upregulated by TBX2 knockdown after 72h as determined by microarray. CoREST factor ZNF217 is indicated in the top 10 enriched terms by the arrowhead. (**B**) Western blot of Co-IP in MCF7 and BT474 cells. ZNF217 was precipitated from lysates using anti-ZNF217 antibody, with species-matched IgG as control IP. Samples were immunoblotted for ZNF217, TBX2, CoREST positive controls (LSD1, HDAC1) and negative control (p53). (**C**) Western blot of Co-IP in BT474 cells. TBX2 was precipitated from lysates using anti-TBX2 antibody, with species-matched IgG as control IP. Samples were immunoblotted as described above. (**D**) MCF7 cells were treated with two independent ZNF217 siRNAs or scrambled control for 72 h. Effects on proliferation were measured by relative cell number; impact on protein levels of ZNF217 and NDRG1 were detected by western blot; effects on mRNA of NDRG1 and CST6 were assessed by RT-qPCR. (**E**) Western blot from MCF7 lysates following treatment with TBX2/LSD1/ZNF217 siRNA for 72 h. Samples were probed with antibodies against each target, to assess mutual dependency for protein expression; effects on NDRG1 upregulation are shown. (**F**) BT474 and T47D cells were treated with ZNF217 siRNA (equimolar pool of sequence#1/sequence#2) or scrambled control for 96h. Effects on protein level of ZNF217 and NDRG1 were detected by western blot; clonogenic assays were performed at 4 weeks (BT474) and 3 weeks (T47D), with effects on colony density shown in representative images and adjacent bar chart. (**G**) MCF7 were treated with 1 μM Entinostat (Entino), 250 nM SP-2509 or a combination of both, with DMSO serving as negative control. Effects on proliferation were measured by relative cell number at 72h; impact on mRNA expression of NDRG1 and CST6 was assessed by RT-qPCR at 48 h. Error bars represent mean ± s.e.m. of three independent experiments. *P< 0.05; **P< 0.01; ***P< 0.001; ns = not significant. GAPDH serves as loading control for all western blots.

The finding that SP-2509 proved our only effective LSD1 inhibitor capable of de-repressing TBX2 target genes was intriguing, as its mechanism of action differs from the classic tranylcypromine-based compounds. Rather than binding to FAD, SP-2509 was shown to disturb physical contact between LSD1 and ZNF217, which normally interact strongly in complexes such as CoREST (39). ChEA enrichment analysis of upregulated transcripts following TBX2 knockdown from an in-house microarray highlighted ZNF217 as the second most significant TF binding site signature (Figure 4A). We therefore surmised that TBX2 may interact with ZNF217, in addition to LSD1 and potentially other members of the CoREST complex. Accordingly, ZNF217 was immunoprecipitated from MCF7 and BT474 cell extracts, which confirmed a physical interaction with TBX2, in addition to the CoREST components LSD1 and HDAC1 (Figure 4B). This was demonstrated in the reciprocal orientation by pulldown of TBX2, showing positive interactions with ZNF217, LSD1 and HDAC1 (Figure 4C), with TBX2-ZNF217 association particularly sensitive to complex disruption with SP-2509 (Supplementary Figure S2). Importantly, these data reproduced previous findings that TBX2 could interact with HDAC1, which was shown to be dependent on the TBX2 C-terminus (3).

The importance of ZNF217 for survival of TBX2-dependent cells was assessed by treatment with two independent ZNF217 siRNA sequences, which resulted in significant growth reduction and transcriptional upregulation of targets NDRG1 and CST6 (Figure 4D). As seen with LSD1, NDRG1 upregulation following ZNF217 knockdown occurred independently of changes to TBX2 protein expression and *vice versa* (Figure 4E). This dependency was further confirmed in BT474 and T47D breast cell lines, wherein knockdown of ZNF217 resulted in marked NDRG1 upregulation and significantly diminished clonogenic survival (Figure 4F). The most obvious cell cycle changes observed with short term knockdown of TBX2, LSD1 or ZNF217 was increased sub-G0, indicative of apoptosis (Supplementary Figure S3). All three proteins were indeed confirmed to be enriched at the NDRG1 promoter by ChIP assay, confirming that a TBX2-CoREST complex was responsible for the repression of TBX2 target genes (Supplementary Figure S4). TBX2 target genes (NDRG1, CST6) did not show consistent upregulation in other TBX2 non-expressing breast cancer lines following knockdown of TBX2, LSD1 or ZNF217, further confirming the requirement for TBX2 in recruiting the CoREST complex to promoters (Supplementary Figure S5).

To further assess CoREST dependency, sensitivity to class I HDACs was confirmed by treating TBX2-expressing cells with increasing doses of inhibitor MS-275 (Entinostat) for 72h, which decreased proliferation with IC50 of 1.5 μM and 0.5 μM in MCF7 and T47D cells, respectively (Supplementary Figures S6A and S6B, respectively). As the disruption of several CoREST-related proteins could relieve repression of TBX2 targets, it was proposed that simultaneous inhibition of multiple complex members should result in a pronounced phenotype. Accordingly, we found that low doses of SP-2509 and Entinostat (IC50 or less) exhibited an effect on growth arrest and transcriptional upregulation of NDRG1/CST6, which was significantly greater than single-agent treatments (Figure 4G). Together these results inferred that the interaction and coordinated activity between TBX2 and CoREST proteins LSD1, ZNF217 and HDAC1 may be essential for repression of TSGs to promote breast tumour survival.

### Global DNA binding of TBX2 varies significantly with tissue context

To better define the mechanisms underpinning TBX2 transcriptional repression, we performed TBX2 ChIP-seq in MCF7 cells with two biological replicates. Peak calling with MACS2 revealed reproducible TBX2 enrichment at >1500 genomic regions (Supplementary Figure S7A). TBX2 peaks were predominantly located at proximal promoters, with *WDR74* identified as the most strongly enriched binding site of known promoters (Supplementary Figure S7B). TBX2 binding was also observed at the NDRG1 promoter, in validation of previous ChIP-PCR work performed by our group (Supplementary Figure S7C). Gene set enrichment analysis of TBX2-bound promoters against databases including MSigDB and NCI-Nature found ‘mTORC1 signalling’, ‘ErbB1 downstream signalling’, ‘unfolded protein response’, ‘PDGFR-beta signalling’ and ‘p53 pathway’ to be among the most significantly enriched terms. In addition, TBX2-targeted promoters exhibited strong enrichment for genes encoding proteins which physically interact with CDK1 and ESR1, further reinforcing the role of TBX2 in regulation of cell cycle progression and differentiation (Supplementary Figure S7D). STREME motif analysis of sequences centred on TBX2 peak summits showed no enrichment for the canonical TBX2 motif AGGTGTGAR, which was anticipated due to previous findings inferring indirect DNA contact via interaction with other transcription factors in breast cancer (11,13). Rather the most strongly enriched consensus sequence was GGGGCGGGGC corresponding to Sp1, followed by centrally enriched motif signatures of KLF12, NF-Y, CTCF, CREM, EGR1, THAP11 and GABPA (Supplementary Figure S7E). Over-representation of the EGR1 motif was compelling, given we previously found that TBX2 interacts with EGR1 to access the NDRG1 promoter via an overlapping Sp1/EGR1 consensus sequence (11). Over 85% of the TBX2 sites containing each motif were located at promoters, with the exception of CTCF; approximately 40% of TBX2 sites containing a CTCF motif were instead located at intragenic and intergenic regions (Supplementary Figure S7E; Table S4). This may be attributed to core functions of CTCF in mediating long-range chromatin interactions, including those between transcription start sites and distal enhancers (40). Taken together, these data indicated that TBX2 predominantly regulates transcription at proximal promoters, however, its physical engagement with DNA at these regions likely occurs indirectly through interaction with key transcription factors such as Sp1.

As our in-house ChIP-seq suggested that TBX2 DNA binding occurs indirectly in breast cancer, we hypothesized that global chromatin occupancy may vary considerably between tissue types with differential expression of TBX2-interacting TFs. This was addressed *in silico* through comparison of our MCF7 data with publicly available TBX2 ChIP-seq in N-Myc amplified neuroblastoma, the only other known cancer in which TBX2 ChIP-seq has been performed to date (4,10). We compared our MCF7 TBX2 ChIP-seq with TBX2 ChIP-seq of the Kelly cell line from the Durbin Zimmerman Dharia et al. study, as this data was of high quality and performed with the same TBX2 antibody (ab33298) (10). Intriguingly the majority of TBX2 sites identified in MCF7 did not exhibit significant TBX2 binding in Kelly, while the inverse was true for the Kelly dataset; overall only 222 overlapping sites exhibited strong TBX2 binding in both MCF7 and Kelly cells (Supplementary Figure S8A). As expected, TBX2 sites in MCF7 which were either lacking in Kelly cells, or overlapping with Kelly cells, were predominantly located at promoter regions. TBX2 sites which were unique to Kelly cells, however, exhibited a strikingly different profile with the majority of binding falling into intragenic and intergenic regions (Supplementary Figure S8B), indicative of the strong participation of TBX2 in deregulated enhancer circuitry of N-Myc neuroblastoma previously described (4,10). Kinase enrichment analysis of promoters bound by TBX2 in both cell lines found EGFR downstream targets to be specific to MCF7, while promoters of genes regulated by CDK7 were specific to the Kelly cell line; this was fitting given that CDK7 is known to be a key driver of N-Myc-dependent super-enhancer activity in tumours with N-Myc amplification (41) (Supplementary Figure S8C). Furthermore, extraction of DNA motifs underlying TBX2 summits in the two cell models found bias for CTCF and NF-Y signatures to be specific to MCF7; in the MCF7/Kelly overlapping regions enrichment for the Sp1 motif was observed, albeit at lower significance due to a smaller number of sampled sequences (Supplementary Figure S8D). In contrast TBX2-bound regions specific to Kelly cells were most strongly centrally enriched for the AP-2 motif, in addition to motifs for previously described neuroblastoma factors PHOX2B, GATA3 and ISL1. Overall these comparisons provide strong evidence for a tissue-specific model of TBX2 engagement with chromatin, mediated indirectly through interaction with core TFs which may vary significantly between cancer subtypes.

### TBX2 and ZNF217 exhibit both distinct and overlapping global DNA binding profiles

**Figure 5.**
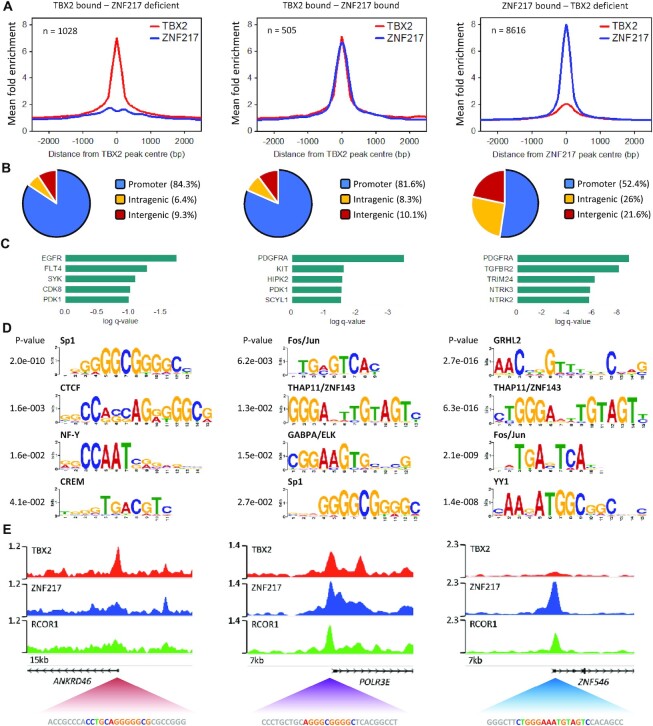
ZNF217 overlaps with one third of global TBX2 binding sites in breast cancer cells. (**A**) TBX2 ChIP-seq conducted in-house on MCF7 cells, compared with public MCF7 ChIP-seq for ZNF217 (ENCODE). Profile plots denote mean enrichment of ChIP read density over input control read density ±2.5 kb for all peak summits consistent between two biological replicates. Three analysis groups of regions were identified corresponding to TBX2 sites lacking significant ZNF217 signal, TBX2 sites overlapping with ZNF217, and ZNF217 sites lacking significant TBX2 signal. (**B**) Distribution of ChIP binding sites between promoter and non-promoter regions corresponding to above analysis group. (**C**) Enrichment q-values for top 5 upstream GEO kinase terms associated with genes from bound promoter regions, corresponding to above analysis group. (**D**) Position weight matrices (PWMs) of significantly enriched TF motifs concentrated within 100bp of ChIP peak summits as identified by STREME, corresponding to above analysis group. List for ZNF217 sites lacking TBX2 signal is truncated to top 4 PWMs; also contains motifs for GABPA/ELK, CTCF, ESR and TEAD4. (**E**) Genome browser snapshots of ChIP-seq tracks representing pooled reads from two biological replicates for each factor. Example promoter regions from each analysis group are shown, with inclusion of public MCF7 RCOR1 ChIP data and exact motif sequence in peak centre highlighted. Scale bars correspond to reads per million.

Based on our evidence that TBX2 resides in a repression complex alongside CoREST factors, we further compared our in-house TBX2 data with publicly available MCF7 ChIP-seq for key proteins in this complex. Due to lack of high-quality LSD1 public data and our inability to achieve LSD1 enrichment with a formaldehyde protocol, we utilized ZNF217 ENCODE ChIP-seq data to represent potential CoREST-targeted regions. We found that ∼30% of TBX2 binding sites overlapped centrally with ZNF217 peaks, with the mean signal enrichment of these peaks highly similar for both proteins (Figure 5A). TBX2 peaks overlapping with ZNF217 were seen primarily at promoter regions (Figure 5B; Supplementary Table S3), which kinase enrichment analysis showed to be enriched for PDGFRA downstream target genes (Figure 5C). Interestingly, these TBX2/ZNF217 overlapping peaks were lacking in enrichment for previously found CTCF and NF-Y motifs, while displaying significant enrichment for Fos/Jun (AP-1), THAP11, GABPA and Sp1 consensus sequences (Figure 5D). In contrast the remaining TBX2 sites deficient in ZNF217 signal were enriched for Sp1, CTCF, NF-Y and CREM motifs but not AP-1, THAP11 or GABPA; taken together this implied that ZNF217 influences localisation of TBX2 to AP-1, THAP11 and GABPA sites, while itself infrequently occurring at TBX2-bound NF-Y, CTCF and CREM sites. Accordingly, the analysis of ZNF217-bound sites deficient in TBX2 signal found significant occurrences of THAP11, AP-1, GABPA and CTCF motifs but no enrichment for NF-Y or CREM sequences (Figure 5D). We further examined TBX2 and ZNF217 binding sites with the inclusion of ENCODE public ChIP-seq data for RCOR1, a key component of the CoREST complex. Target genes showing enrichment of all three proteins would represent likely TBX2-CoREST repressed genes. While a subset of TBX2/ZNF217 shared regions displayed overlapping RCOR1 signal, no central RCOR1 enrichment was observed in remaining TBX2 sites deficient in ZNF217 binding (Supplementary Figure S9; Figure 5E). Indeed, we observed over 1000 genomic sites where TBX2 bound independently of ZNF217 (listed in Supplementary Table S2). Overall, these data highlighted that ZNF217 was essential for RCOR1 localisation to TBX2 target regions, and uncovered a select group of target promoters whereby TBX2 may interact with CoREST proteins to achieve transcriptional repression. The importance of ZNF217 for TBX2-CoREST repression may also explain why disruption of ZNF217 interactions within the complex by SP-2509 was effective in de-repressing TSGs and inhibiting breast cancer cell proliferation.

### Sp1 is essential for TBX2-mediated repression of NDRG1

**Figure 6.**
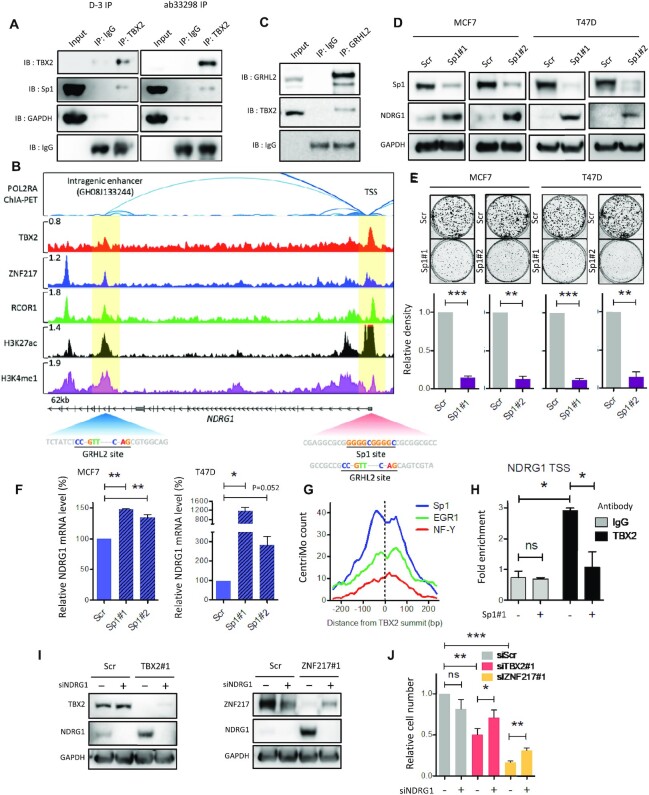
Sp1 is required for repression of TSG NDRG1 by recruiting TBX2 to the NDRG1 promoter. (**A**) Western blot of Co-IP in MCF7 cells. TBX2 was precipitated from lysates using either a mouse monoclonal (D-3) or rabbit polyclonal (ab33298) antibody, with species-matched IgG as control IP. Samples were immunoblotted for TBX2 and Sp1, with GAPDH serving as negative control. (**B**) Genome browser snapshot detailing regulation of the *NDRG1* locus. All tracks represent 2 biological replicates of MCF7 data for POL2RA ChIA-PET (ENCODE), TBX2 ChIP-seq (in-house), ZNF217/RCOR1 ChIP-seq (ENCODE), and ChIP-seq for enhancer histone marks (ENCODE). Physical association between the highlighted regions is indicated by ChIA-PET arcs above, with GeneHancer catalogue number included. Key TF motifs at each region are highlighted below, with fully conserved Sp1 sequence visible within TBX2 peak centre at the promoter/TSS. Scale bars correspond to reads per million. (**C**) Western blot of Co-IP in MCF7 cells. GRHL2 was precipitated from lysates using anti-GRHL2 antibody, with species-matched IgG as control IP. Samples were immunoblotted for GRHL2 and TBX2. (**D**) Western blot from lysates following treatment with Sp1 siRNAs for 120 h (MCF7) and 96h (T47D). Samples were probed with anti-Sp1 and anti-NDRG1 antibody. (**E**) Clonogenic assays following treatment with Sp1 siRNAs for 2 weeks (MCF7) and 3 weeks (T47D); effects on colony density are shown in representative images and below bar charts. (**F**) RT-qPCR of MCF7 and T47D cDNA time-matched to above western blot experiments, assessing mRNA expression of NDRG1 with Sp1 siRNA vs scrambled control. (**G**) CentriMo distribution plot of motifs specific to MCF7 TBX2 ChIP-seq in this study. Chart represents frequency counts of best motif match, relative to peak summit and totalled from all peak sequences where a match was found (counts binned at 20bp to improve resolution). Sp1 motif indicated as most frequently occuring, with matches concentrated in central region proximal to TBX2 binding site. (**H**) ChIP-PCR using anti-TBX2 antibody (ab33298) or species-matched IgG control in MCF7 cells treated with scrambled/Sp1 siRNA for 120h. PCR signals spanning the NDRG1 TSS and nonspecific region were calculated as % input; relative binding of TBX2 was determined by fold enrichment of PCR signal at TSS versus nonspecific region. (**I**) Western blot from MCF7 lysates following treatment with TBX2 or ZNF217 siRNA, in the presence of scrambled (−) or NDRG1 (+) siRNA for 96 h. Samples were probed with antibodies against TBX2, ZNF217 and NDRG1. (**J**) Relative cell number time-matched to siNDRG1 western blot experiments. Error bars represent mean ± s.e.m. of three independent experiments (ChIP-PCR represents two biological replicates). *P< 0.05; **P< 0.01; ***P< 0.001; ns = not significant. GAPDH serves as loading control for all western blots.

Following completion of TBX2 ChIP-seq in MCF7 we wanted to elucidate how repression of the NDRG1 tumour suppressor was achieved, given our historical interest in this gene as a key target of TBX2 (11,15). Given that TBX2 binding sites from MCF7 ChIP-seq were most strongly enriched for the Sp1 motif (Supplementary Figure S7E), we hypothesised that Sp1 may be integral to TBX2-mediated gene repression. Accordingly, immunoprecipitation experiments confirmed that TBX2 interacted with Sp1 (Figure 6A). We next examined the NDRG1 locus for occurrences of Sp1 recognition sites (Figure 6B). In comparison to other TBX2/CoREST targets identified *in silico*, the arrangement of TBX2, ZNF217 and RCOR1 binding sites at the *NDRG1* locus followed an atypical pattern; while clear TBX2 and RCOR1 peaks were present at the promoter/TSS, this region lacked a strong ZNF217 peak. Instead ZNF217 binding was found to be enriched at intronic regions at the 3’ end of the *NDRG1* locus, co-occupied by RCOR1. Importantly, the region at intron 10 was classed as an ‘intragenic enhancer’, as catalogued in the GeneHancer double elite database to target the NDRG1 TSS (Figure 6B). Evidence for intron 10 as an internal enhancer for *NDRG1* was further corroborated by public MCF7 data, showing this region physically interacted with the promoter in 3D assays; both the promoter and intron 10 were also enriched for histone modifications H3K27Ac and H3K4me1, the hallmarks of active enhancers (42). As such, lack of strong ZNF217 signal at the NDRG1 promoter could be explained by chromatin looping, influencing the fixation of proteins from one complex to two apparently distinct DNA regions. Although our evidence indicated that NDRG1 was a TBX2-CoREST repression target, we expected a degree of active histone modification to be present at this locus due to basal expression of NDRG1 in the MCF7 cell line (Supplementary Figure S6A). Recent work found that internal enhancers may in fact be repressive in nature, by interfering with basal transcription of their respective host genes (43). Interestingly, motifs for the master epithelial factor GRHL2 (CCNGTTNNNCNAG) were present in ChIP peak centres at both the *NDRG1* promoter and at intron 10 (Figure 6B), while a fully conserved Sp1 sequence (GGGGCGGGGC) was specific to the centre of the TBX2 peak at the promoter. These observations were fitting, given previous findings that GRHL2 and Sp1 were key motifs involved in global binding of ZNF217 and TBX2, respectively, in MCF7 cells (Figure 5D). Interestingly, TBX2 was found to physically interact with GRHL2 (Figure 6C). However, knockdown of Sp1 alone was seen to be sufficient for NDRG1 upregulation in TBX2-dependent cell lines, as assessed by western blot (Figure 6D), while the loss of this protein significantly diminished long-term survival (Figure 6E). Analysis of matched mRNA also confirmed that NDRG1 upregulation following Sp1 loss occurred at a transcriptional level (Figure 6F); as such these data implied that Sp1 may be important for NDRG1 repression and maintenance of cellular proliferation. Review of our TBX2 ChIP-seq data found that Sp1 motifs were predominantly located within 100bp of TBX2 peak summits, indicative of cooperative or competitive DNA binding between these two factors (Figure 6G). In the case of the *NDRG1* locus, ChIP PCR demonstrated a significant loss of TBX2 binding to the promoter/TSS region following Sp1 knockdown (Figure 6H). Indeed, knockdown of Sp1 also reduced ZNF217 and LSD1 recruitment (Supplementary Figure S10). Having previously demonstrated the important role of NDRG1 repression in maintaining cell proliferation (11), we observed that siRNA knockdown of NDRG1 (Figure 6I) partially rescued the antiproliferative effect of TBX2 and ZNF217 knockdowns (Figure 6J). Together these findings provided evidence that transcriptional repression of NDRG1 by the TBX2-CoREST complex is achieved via recruitment of TBX2 to the NDRG1 promoter by Sp1. While NDRG1 was contributing to tumour suppression following loss of TBX2/CoREST, a number of other target genes (potentially novel TSGs) were likely involved in this phenotype and therefore warranted further investigation.

### LINC00111 is repressed by TBX2-CoREST and exhibits tumour-suppressive activity in breast cancer cells

**Figure 7.**
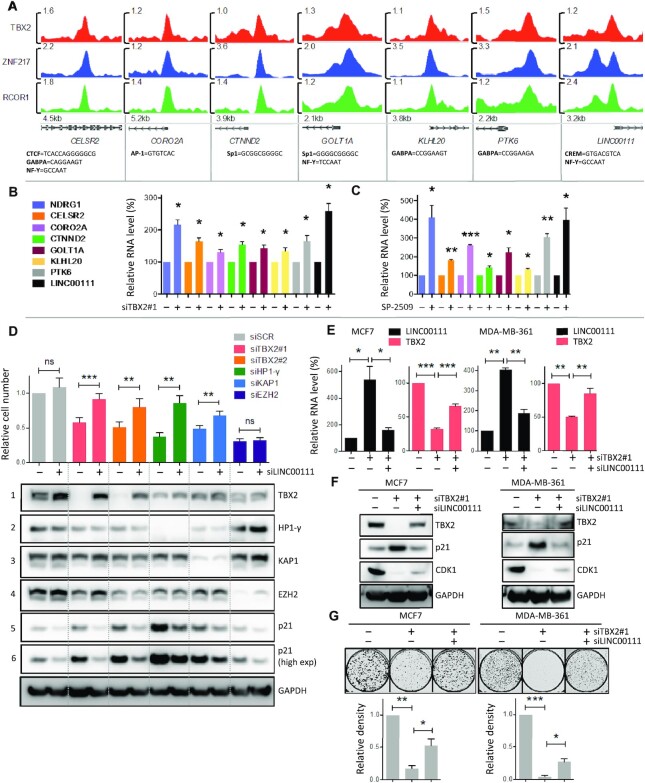
Long non-coding RNA LINC00111 is a transcriptional repression target of TBX2-CoREST which exhibits tumour-suppressive activity. (**A**) Genome browser snapshots representing two biological replicates of TBX2, ZNF217 and RCOR1 ChIP-seq in MCF7 cells; regions indicate bound promoters of interest, of which transcripts were confirmed by PCR as repression targets of TBX2 and CoREST. Exact motif sequences found in peak centres are listed below and scale bars correspond to reads per million. (**B**) RT-qPCR of MCF7 cells treated with scrambled (−) or TBX2 (+) siRNA for 72 h, assessing upregulation of candidate TBX2-CoREST targets as discovered by ChIP-seq. NDRG1 serves as a positive control. (**C**) RT-qPCR of MCF7 cells treated with DMSO (−) or 250nM SP-2509 (+) for 72h, assessing upregulation of candidate target genes as described above. NDRG1 serves as a positive control. (**D**) Relative cell counts and matched western blot of MCF7 cells treated with TBX2/ HP1-γ/KAP1/EZH2 siRNA, in the presence of scrambled (−) or LINC00111 (+) siRNA for 96h. Western columns correspond to above condition in bar graph; each antibody panel consists of a single exposure of a single image, with overlaid dashed lines separating samples into each TF siRNA treatment group. (**E**) RT-qPCR of MCF7 and MDA-MB-361 cells treated with scrambled siRNA (−/−), or TBX2 siRNA in the presence of scrambled (+/−) or LINC00111 (+/+) siRNA for 96h. Primers were used to assess RNA expression changes in LINC00111 and TBX2 as indicated. (**F**) Western blot of MCF7 and MDA-MB-361 lysates time-matched to above RT-qPCR experiments; antibodies were used to assess protein expression of TBX2, senescence marker p21^WAF1/CIP1^ and proliferation marker CDK1. (**G**) Clonogenic assays following treatment with scrambled/TBX2/LINC00111 siRNA as described above for 2 weeks (MCF7) and 4 weeks (MDA-MB-361); colony density measurements are shown in bar charts in order of the above representative images. Error bars represent mean ± s.e.m. of three independent experiments. *P< 0.05; **P< 0.01; ***P< 0.001; ns = not significant. GAPDH serves as loading control for all western blots.

Given the large proportion of TBX2 binding sites occupied by ZNF217 in MCF7 ChIP-seq, we determined which of these regions represented TBX2-CoREST-repressed genes that may function as tumour suppressors. Of the subset of TBX2/ZNF217 bound regions containing RCOR1, we shortlisted seven promoters of which transcripts were consistently upregulated by loss of TBX2 or ZNF217 function; 6 of these genes were protein-coding (*CELSR2*, *CORO2A*, *CTNND2*, *GOLT1A*, *KLHL20*, *PTK6*) and one gene represented a long non-coding RNA product (*LINC00111*) (Figure 7A). Each of these genes contained at least one of the motifs previously found as significant, within the TF binding site (Figure 5D). The *LINC00111* promoter was distinct from other regions as the TBX2 peak contained a CREM motif, which was less commonly-occurring in TBX2 binding sites overall (Figure 7A; Supplementary Table S4). We found that each of these seven genes were transcriptionally repressed by TBX2-CoREST, as treatment with TBX2 siRNA or ZNF217/LSD1 inhibitor SP-2509 resulted in significant upregulation of the associated RNA. LINC00111 was found to be the most strongly upregulated transcript, with fold RNA induction comparable to NDRG1 (Figure 7B and C).

As the function of LINC00111 was unknown, we determined whether this TBX2-CoREST-repressed target played a role in cellular growth and survival. To address this question, TBX2 was knocked down in the absence and presence of LINC00111 siRNA, with effects on cell growth compared (Figure 7D). Knockdowns of HP1-γ, KAP1 and EZH2 were also included, as we previously found these factors important for TBX2-mediated TSG repression and cell proliferation (15). We found that LINC00111 siRNA treatment significantly rescued the antiproliferative effects of TBX2 siRNAs, as well as HP1-γ and KAP1 knockdown (Figure 7D). Interestingly, treatment with LINC00111 siRNA alone led to marked upregulation of TBX2 protein, which naturally opposed the effects of TBX2 siRNA (Figure 7D, panel 1); this implied that LINC00111 may itself behave as a repressor of TBX2. In addition, LINC00111 siRNA basally reduced protein levels of pro-senescence factor p21^WAF1/CIP1^, while preventing p21^WAF1/CIP1^ upregulation by TBX2 siRNA (Figure 7D, panels 5 and 6). LINC00111 siRNA did not impact on protein expression of HP1-γ or KAP1 (Figure 7D, panels 2 and 3); however, the upregulation of p21^WAF1/CIP1^ by HP1-γ/KAP1 knockdown was greatly reduced by LINC00111 siRNA treatment (Figure 7D, panels 5 and 6). Conversely, LINC00111 siRNA did not rescue the antiproliferative effect of EZH2 knockdown, nor did EZH2 knockdown result in upregulation of p21^WAF1/CIP1^ (Figure 7D, panels 4, 5 and 6). Taken together, these data indicated that LINC00111 played an important role in the context of growth arrest associated with p21^WAF1/CIP1^ induction.

To further confirm the role of LINC00111 in growth arrest, experiments were repeated in MCF7 and MDA-MB-361 cells, wherein TBX2 was knocked down in the absence and presence of LINC00111 siRNA. The upregulation of LINC00111 following TBX2 loss, and subsequent knockdown by LINC00111 siRNA were confirmed by RT-qPCR. As observed previously, LINC00111 knockdown induced upregulation of TBX2 itself (Figure 7E and F). In both cell lines, LINC00111 knockdown prevented p21^WAF1/CIP1^ upregulation by TBX2 siRNA and partially rescued the loss of proliferation marker CDK1 (44) (Figure 7F). In long-term assays, LINC00111 knockdown also significantly reduced the detrimental effect of TBX2 siRNA on colony formation (Figure 7G). Taken as a whole, these results demonstrated that LINC00111 possesses tumour-suppressive properties by regulating both TBX2 and p21^WAF1/CIP1^ expression, and as such has important ramifications for cancer cell survival when targeted for repression by the TBX2-CoREST complex.

## DISCUSSION

**Figure 8.**
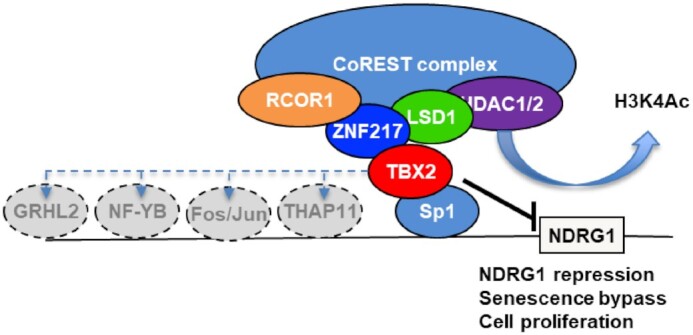
Schematic diagram showing the proposed mechanism of TBX2-CoREST repression of target genes such as NDRG1. TBX2 co-opts numerous transcription factors (TFs), most notably in this case Sp1, to install a CoREST repression complex at target promoters. Demethylation (*via* LSD1) and deacetylation (*via* HDAC1/2) events provided by the CoREST complex then catalyse the closing of chromatin around target promoters, leading to the shutdown of senescence, repression of growth control genes such as NDRG1 and P21^WAF1^, senescence-bypass and maintenance of proliferation. Some of the other leading candidate TF interactors of TBX2 (as predicted through motif searching of TBX2 ChIP-seq data) are shown (adjacent left of Sp1).

Here, we have identified a novel mechanism through which the oncogenic repressor TBX2 interacts with CoREST complex proteins to target growth control and senescence genes, thereby facilitating maintenance of proliferation of breast cancer cells. have We show that TBX2 binding is associated with a wide range of TF motifs, which may be important for the recruitment of the TBX2-CoREST complex, as demonstrated by the interaction with Sp1. A hypothetical model of how this TBX2-CoREST repression complex is organised and how it assembles on target promoters is shown in Figure 8. The role of CoREST in breast biology and indeed cancer pathogenesis, is highly context dependent. For example, Breast Carcinoma Metastasis Suppressor gene 1 (BRMS1) was found to be a core component of the LSD1-CoREST complex, using these interactions to suppress breast cancer migration and invasion (45). A transcription factor, ZNF516, was shown to recruit the CtBP/LSD1/CoREST complex to inhibit the proliferation and invasion of breast cancer cells *in vitro* and suppress breast cancer growth and metastasis *in vivo* (46). Conversely, it has also been shown that the SNAG domain of SNAIL1 functions as a molecular hook to recruit LSD1 to repress epithelial promoters, thus driving EMT in breast cancer (47).

As part of this study we were keen to identify enzymatic regulators associated with TBX2, in particular regulators playing key roles in TBX2 functional repression. The enzymatic activities resident within the CoREST complex have long been an attractive opportunity for drug design. A combined LSD1-HDAC inhibitor, CORIN, a synthetic hybrid agent derived from the Class I HDAC inhibitor (Entinostat) and an LSD1 inhibitor (tranylcypromine analog), showed much greater growth inhibition of melanoma lines compared to Entinostat alone (26). Similarly, we show in this study that combining LSD1 and HDAC inhibitors produced potent growth inhibition and target gene de-repression in TBX2-expressing breast cancer lines. However, what has become obvious from our study and others is that targeting enzymatic activity is not the only method of inhibiting CoREST function, as specific allosteric interactions are now being identified as key modes of regulation. As alluded to previously, the inhibitor GSK-LSD1, a tranylcypromine-based molecule designed to target the demethylase function of LSD1, actually achieves anti-proliferative effects through allosteric inhibition of GFI1B (38). Similarly, the allosteric inhibitor used in this study, SP-2509, has been shown to disrupt the LSD1-ZNF217 interaction and has proven highly effective in inhibiting the proliferation of prostate cancer cells, much more so than ‘catalytic’ inhibitors. The scaffold function of ZNF217 may be particularly important for the identified internal enhancer regulating the *NDRG1* locus (Figure 6B). Our work is strikingly similar to the Sehrawat et al. study, suggesting that the ZNF217-LSD1 association may also be a key interaction within the CoREST complex in TBX2-addicted breast cancers.

In this study we have identified of a host of potential transcription factor interactors of TBX2, which may be reprogrammed by TBX2 to exert anti-senescence and proliferative phenotypes. Amongst these, the SP family is linked to a number of processes such as growth control, DNA damage responses and cell cycle control, with Sp1 being a key activator of p21^WAF1/CIP1^ (48). Indeed, we observe from TBX2 ChIP-seq in MCF7 cells that a well-conserved Sp1/KLF12 motif is present in the centre of its binding site at the p21^WAF1/CIP1^ promoter (Supplementary Table S4); further work would determine whether interaction with Sp1 is necessary for TBX2 recruitment to the p21^WAF1/CIP1^ promoter, as we have shown here with NDRG1 (Figure 6H). THAP11 is a stem cell regulator, responsible for transcription of cell cycle control genes and repression of c-Myc (49,50). Likewise, CREM is considered a TSG, although again tissue specificity may be important in determining whether this factor supports or restrains cell proliferation (51,52). In each case, the reported TSG function of these transcription factors may not only be compromised, but actively targeted for repression by the pro-oncogenic functions of TBX2 and the accompanying CoREST complex. The existence of a CREM motif within the TBX2 binding site at the *LINC00111* promoter is intriguing, given we have shown this gene to be necessary for p21^WAF1/CIP1^ induction and cell cycle arrest following loss of TBX2 function. Additional work would be needed to ascertain whether TBX2-CoREST interact with, or require CREM to gain access to the LINC00111 promoter and achieve transcriptional repression. As the function of LINC00111 is to date unknown, one can only speculate as to how this long non-coding RNA manipulates expression of TBX2 and p21^WAF1/CIP1^. It has however been found that p21^WAF1/CIP1^ expression is regulated by other lncRNAs, such as the p53-regulated gene lincRNA-p21; lincRNA-p21 was seen to function as an activator of the nearby *CDKN1A* gene, by associating with the Heterogeneous Nuclear Ribonucleoprotein K (hnRNP-K) cofactor during p53-dependent p21^WAF1/CIP1^ transcription (53). Mechanism notwithstanding, repression of LINC00111 by TBX2-CoREST appears to be important for breast cancer proliferation and survival; analysis of the METABRIC dataset also indicates that breast cancer patients with LINC00111-over-expressing tumours have markedly better overall survival at months 120–240 of the study, compared to tumours in which LINC00111 is downregulated (Supplementary Figure S11).

Our ChIP-seq study also revealed a large cohort of TBX2 binding regions appearing to be CoREST-independent. For example, ZNF217 overlapped with only 30% of TBX2-bound regions, suggesting that TBX2 associates with a number of other transcription complexes. This is not surprising given that other significant hits were observed in our initial esiRNA screen, including HDAC7, SIRT3 and JMJD2B. HDAC7 has been shown to maintain breast and ovarian cancer stem cells through regulation of H3K27 acetylation at super-enhancer-associated genes (54). The mitochondrial deacetylase SIRT3 was shown to behave as a TSG in breast tissue with SIRT3^–/–^ mice developing breast cancers, while human breast cancers have reduced SIRT3 expression (55). A direct TBX2-SIRT3 interaction would seem unlikely, although SIRT3 has been reported to localise to the nucleus to deacetylate H4K16, a known euchromatin mark (56). It is therefore plausible that TBX2 could reposition SIRT3 to reduce promoter-specific and/or global H4K16 acetylation, a phenomenon which has been reported in multiple tumour types. The histone demethylase JMJD2B/KDM4B is an ERα transcriptional target which is recruited to ERα target sites, to demethylate H3K9me3 and facilitate activation of ERα responsive genes (57,58). However, like LSD1, JMJD2B is also a dual H3K4/K9 demethylase and therefore possesses the ability to both activate and repress transcription, a feature which could also be exploited by TBX2.

TBX2 amplification has been reported to be enriched in BRCA1 mutant breast cancers (which are predominantly ERα-negative) but the *in vitro* models we have used are predominantly ERα-positive. Using the online tool *KM Plotter*, we see that TBX2 mRNA expression (as evaluated using 4 different probesets) correlated (significantly in most instances) with poor outcomes in ERα breast cancers and within the Triple Negative Breast Cancer (TNBC) subtype (Supplementary Figure S12). TNBCs represent the poorest outcome subtype being notoriously hard to treat, highly heterogenous with high metastases rates and in contrast to other breast cancer subgroups, they no current targeted therapies available. These correlations suggest that targeting TBX2-CoREST may represent a novel therapeutic approach for targeting the poorest outcome cancers within the TNBC subtype.

In conclusion, we have identified a novel transcriptional complex formed by the TBX2 oncogenic repressor. The adaptability of TBX2 to repurpose other TFs and interface with multiple repression complexes makes it an extremely potent oncogene. However, by defining the mechanistic insights into how this oncogene initiates tumorigenesis (for example, through senescence bypass or apoptotic thresholding) and how it drives breast cancer pathogenesis, we could exploit real opportunities for the development of specific and effective treatments for TBX2 ‘addicted’, poor outcome breast cancers.

## DATA AVAILABILITY

Data underlying TBX2 ChIP-seq in MCF7 cells is available from GEO under accession number GSE180213 and can be accessed using the token orcbeawunbuzbcz; data can also be viewed interactively using the UCSC browser link below: https://genome.ucsc.edu/s/AmcI91/fy3lbp0sCTFZwA%3D%3D.

## Supplementary Material

gkac494_Supplemental_Files
