# Supplementary material for: TBX2 acts as a potent transcriptional silencer of tumour suppressor genes through interaction with the CoREST complex to sustain the proliferation of breast cancers
Source: Nucleic Acids Res. 2022 Jun 10;50(11):6154–73. doi: 10.1093/nar/gkac494 (PMC9226508; doi:10.1093/nar/gkac494)
Supplement: gkac494_Supplemental_Files [file gkac494_supplemental_files.zip › McIntyre et al TBX2 NAR Supplementary Material Figures Final.pptx]

## Slide 1
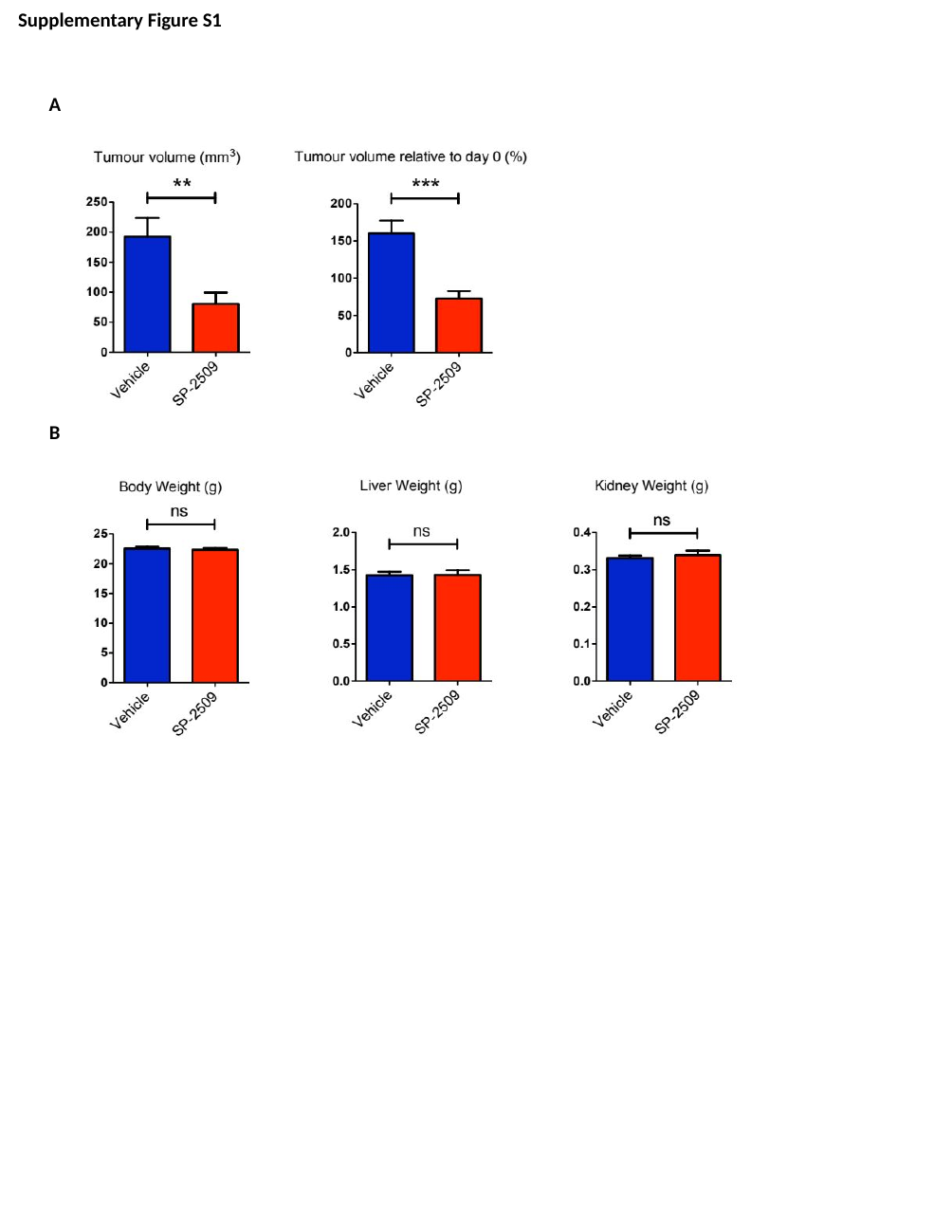

Supplementary Figure S1
A
B

## Slide 2
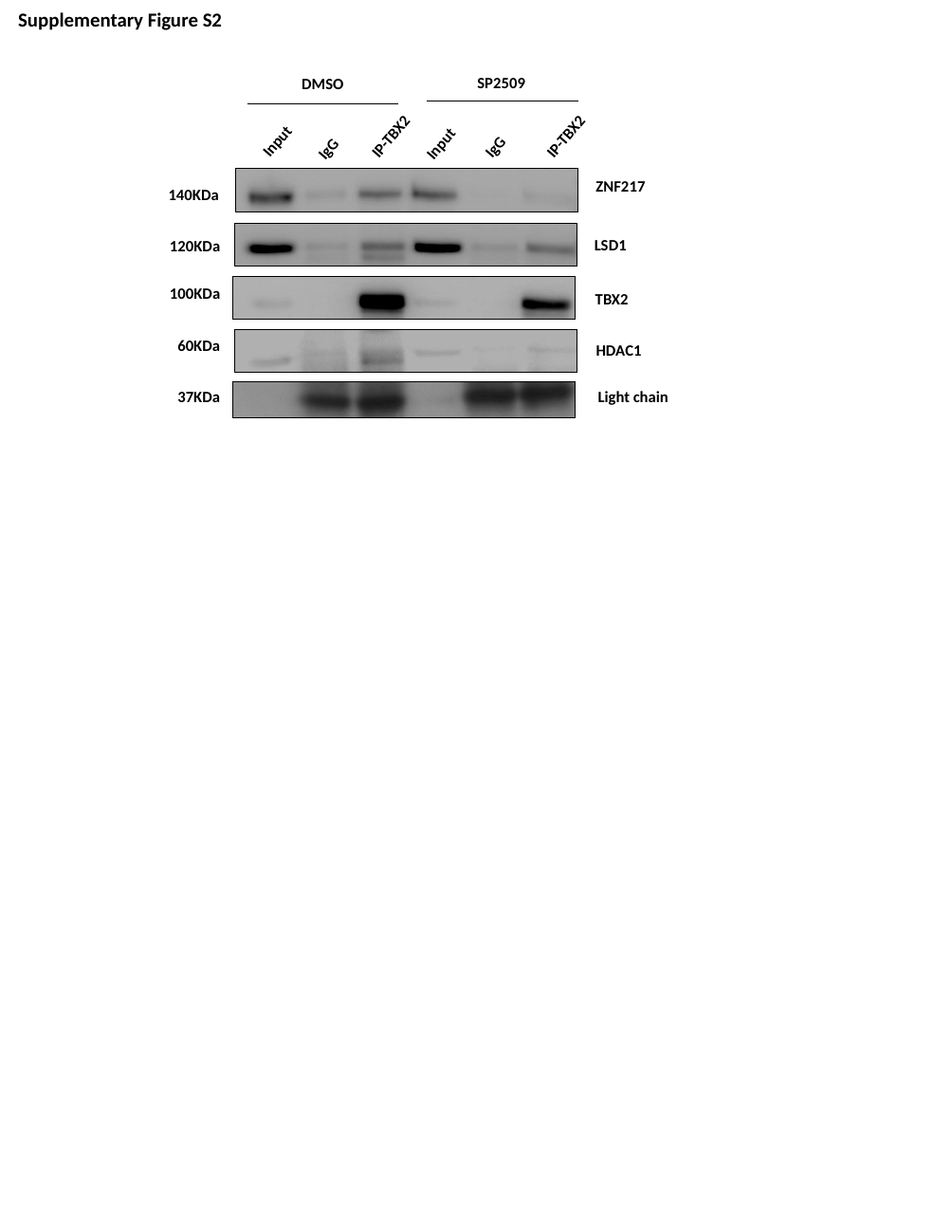

Supplementary Figure S2
SP2509
DMSO
IP-TBX2
IP-TBX2
Input
Input
IgG
IgG
ZNF217
140KDa
LSD1
120KDa
100KDa
TBX2
60KDa
HDAC1
37KDa
Light chain

## Slide 3
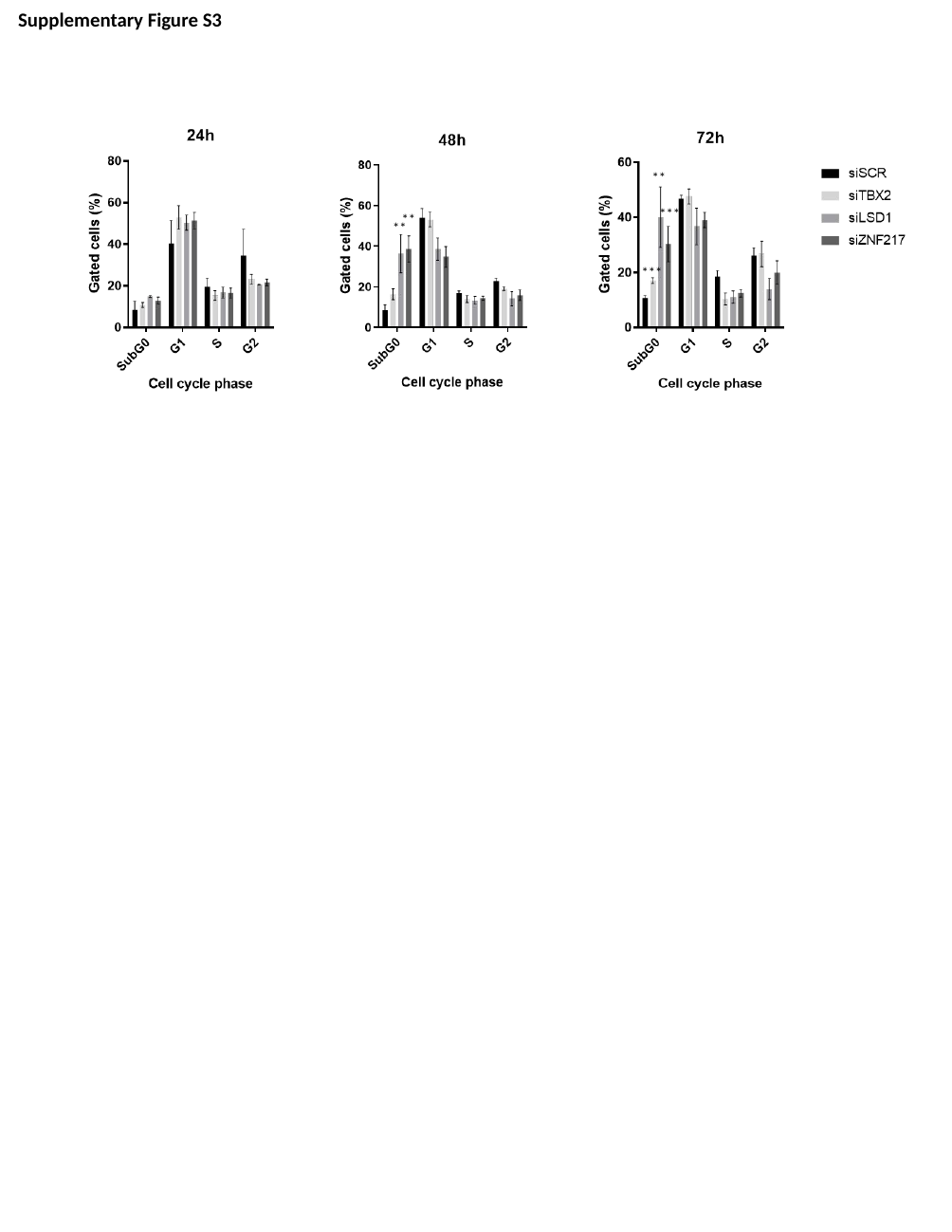

Supplementary Figure S3
**
***
**
**
***

## Slide 4
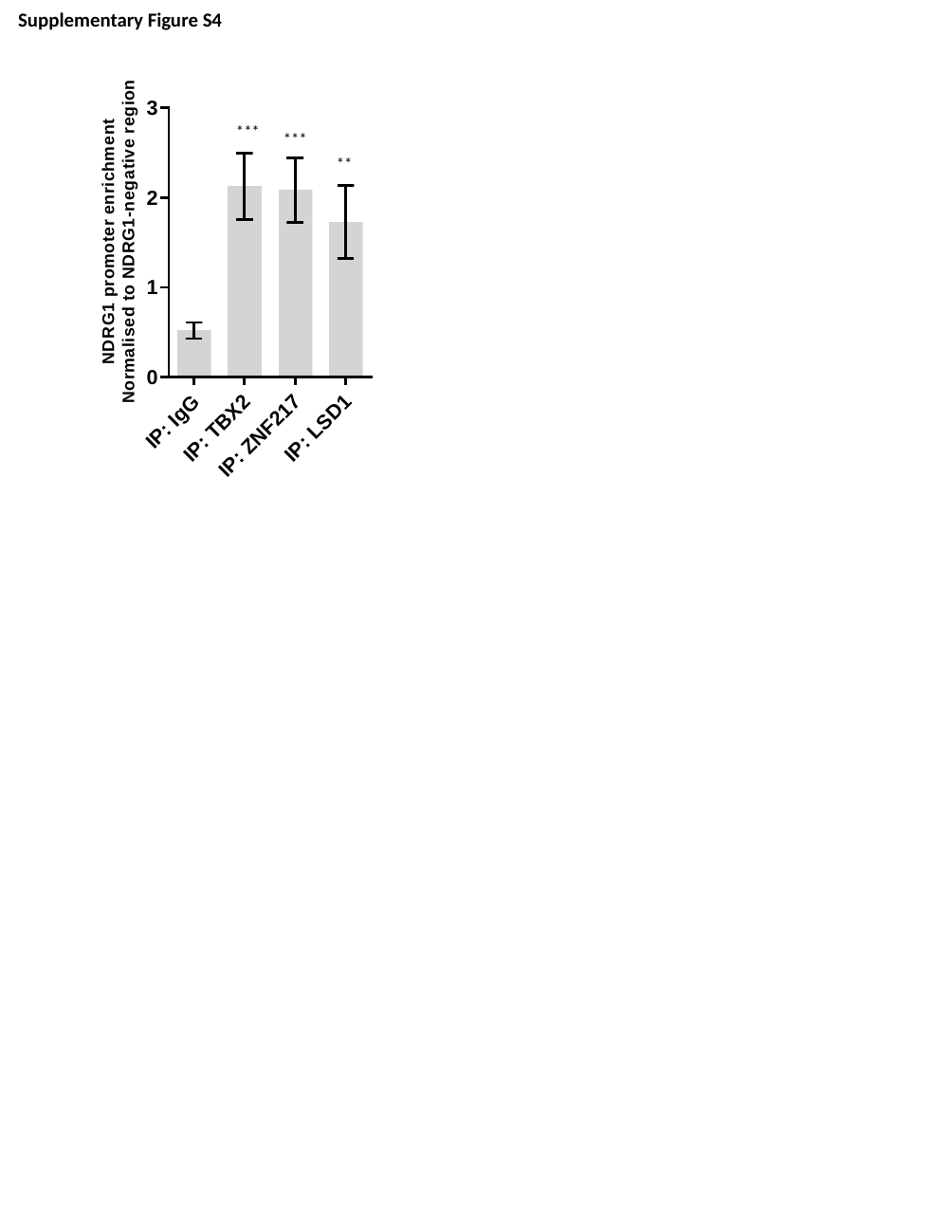

Supplementary Figure S4
***
***
**

## Slide 5
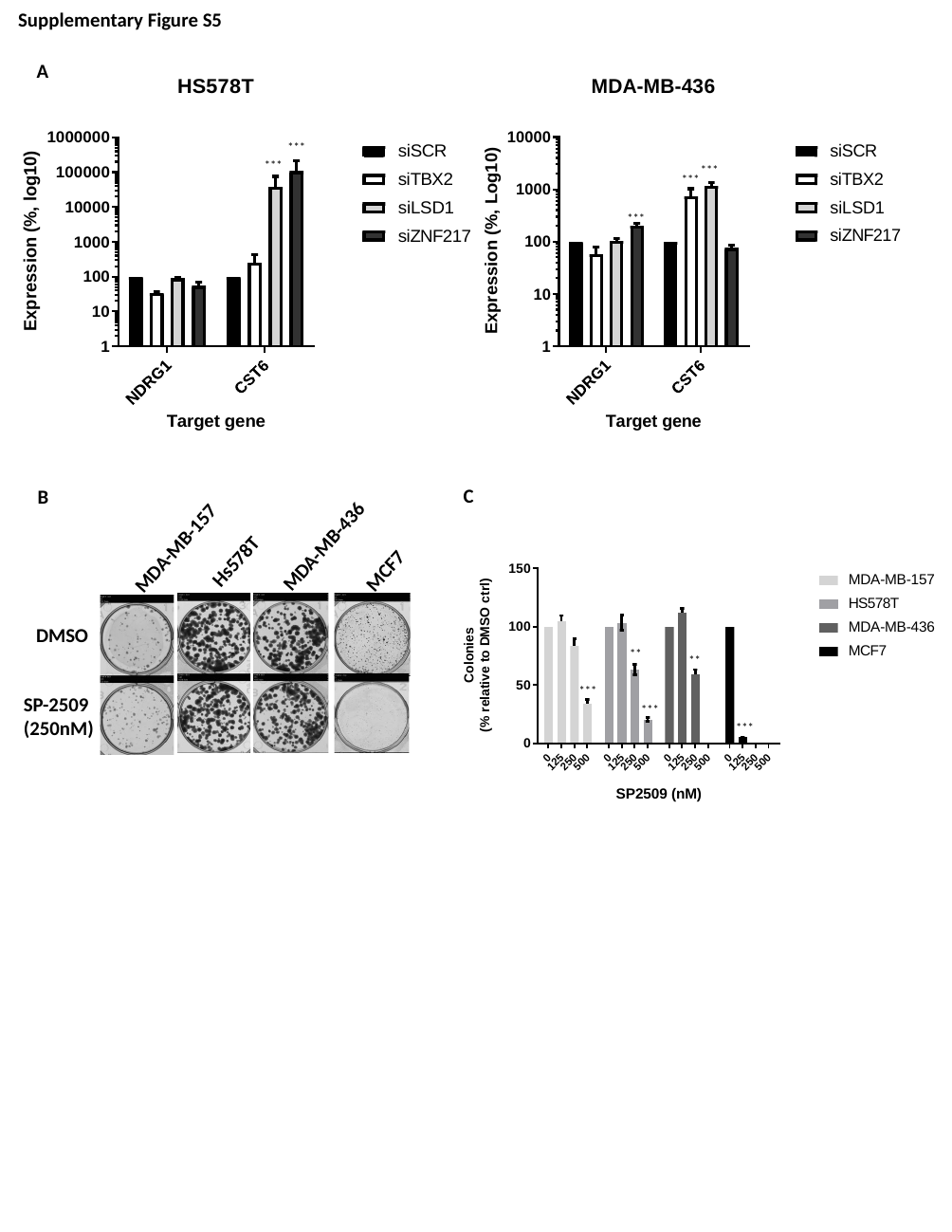

Supplementary Figure S5
A
***
***
***
***
***
C
B
MDA-MB-436
MDA-MB-157
Hs578T
MCF7
DMSO
**
**
***
SP-2509
(250nM)
***
***

## Slide 6
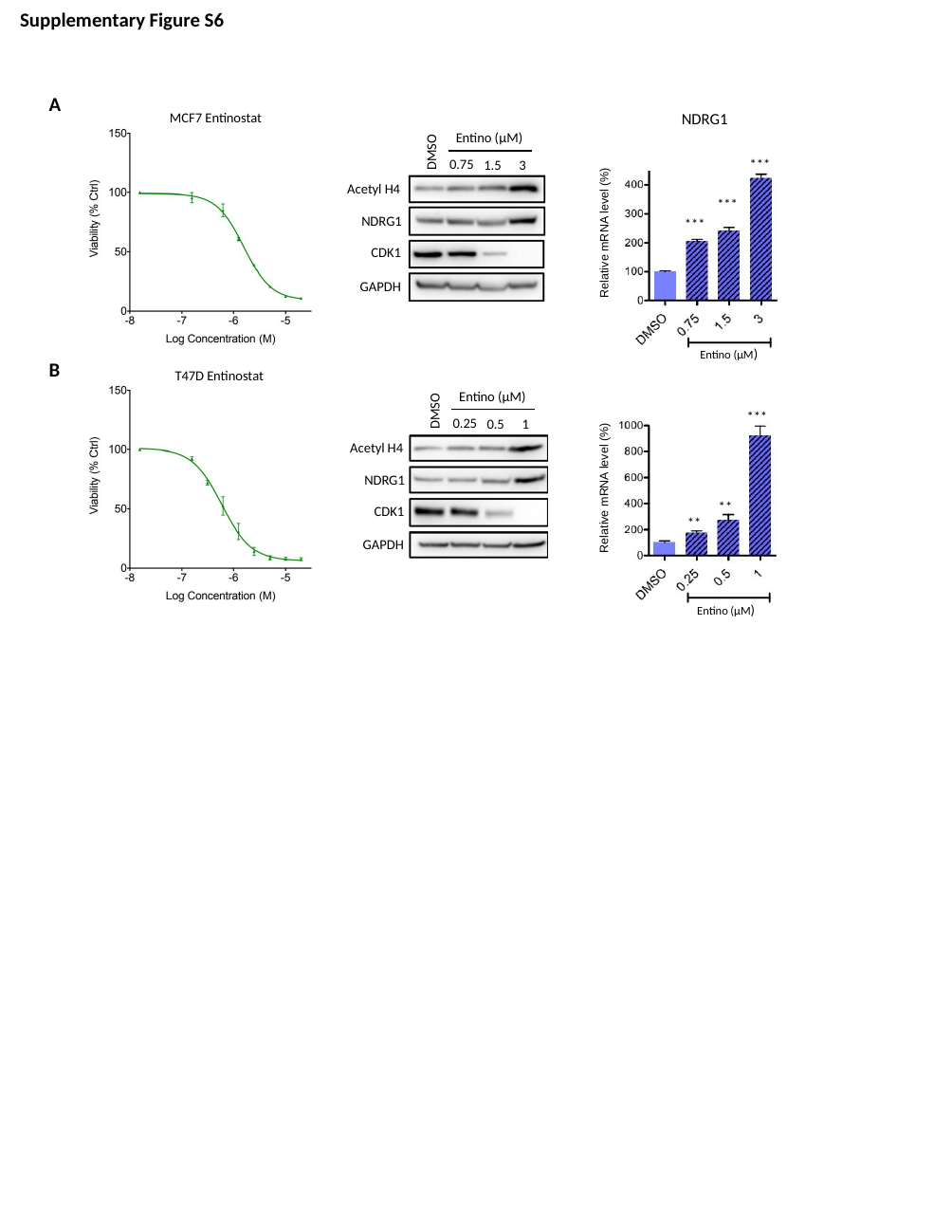

Supplementary Figure S6
A
MCF7 Entinostat
NDRG1
Entino (μM)
DMSO
0.75
***
1.5
3
Acetyl H4
***
NDRG1
***
Relative mRNA level (%)
CDK1
GAPDH
Entino (μM)
B
T47D Entinostat
Entino (μM)
DMSO
***
0.25
0.5
1
Acetyl H4
NDRG1
Relative mRNA level (%)
**
CDK1
**
GAPDH
Entino (μM)

## Slide 7
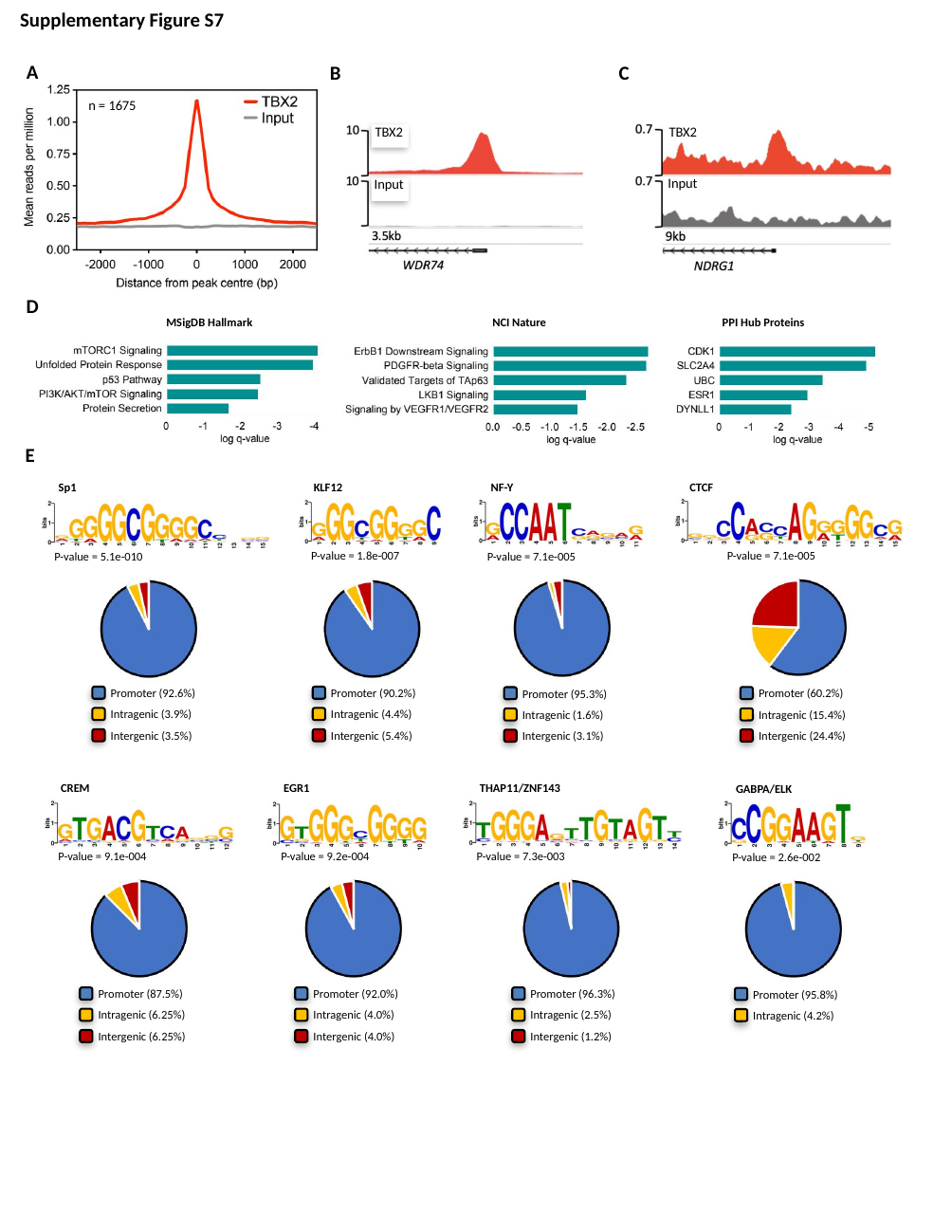

Supplementary Figure S7
A
B
C
n = 1675
TBX2
TBX2
Input
Input
D
MSigDB Hallmark
PPI Hub Proteins
NCI Nature
E
Sp1
CTCF
KLF12
NF-Y
P-value = 1.8e-007
P-value = 7.1e-005
P-value = 5.1e-010
P-value = 7.1e-005
Promoter (92.6%)
Intragenic (3.9%)
Intergenic (3.5%)
Promoter (90.2%)
Intragenic (4.4%)
Intergenic (5.4%)
Promoter (60.2%)
Intragenic (15.4%)
Intergenic (24.4%)
Promoter (95.3%)
Intragenic (1.6%)
Intergenic (3.1%)
CREM
EGR1
THAP11/ZNF143
GABPA/ELK
P-value = 9.1e-004
P-value = 9.2e-004
P-value = 7.3e-003
P-value = 2.6e-002
Promoter (87.5%)
Promoter (92.0%)
Promoter (96.3%)
Promoter (95.8%)
Intragenic (6.25%)
Intragenic (4.0%)
Intragenic (2.5%)
Intragenic (4.2%)
Intergenic (6.25%)
Intergenic (4.0%)
Intergenic (1.2%)

## Slide 8
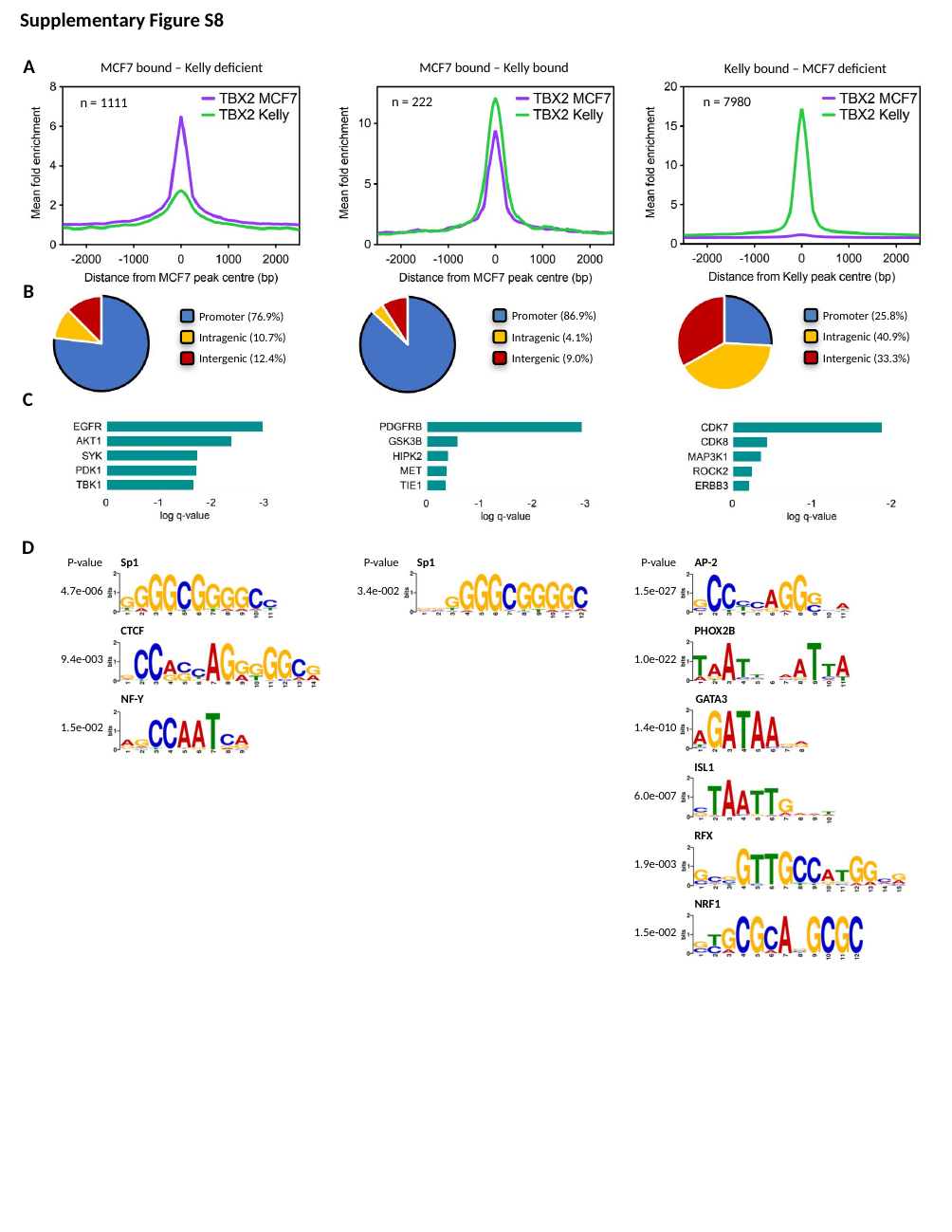

Supplementary Figure S8
A
MCF7 bound – Kelly bound
MCF7 bound – Kelly deficient
Kelly bound – MCF7 deficient
n = 7980
n = 222
n = 1111
B
Promoter (25.8%)
Promoter (86.9%)
Promoter (76.9%)
Intragenic (40.9%)
Intragenic (4.1%)
Intragenic (10.7%)
Intergenic (33.3%)
Intergenic (9.0%)
Intergenic (12.4%)
C
D
P-value
P-value
P-value
Sp1
Sp1
AP-2
4.7e-006
3.4e-002
1.5e-027
CTCF
PHOX2B
9.4e-003
1.0e-022
GATA3
NF-Y
1.4e-010
1.5e-002
ISL1
6.0e-007
RFX
1.9e-003
NRF1
1.5e-002

## Slide 9
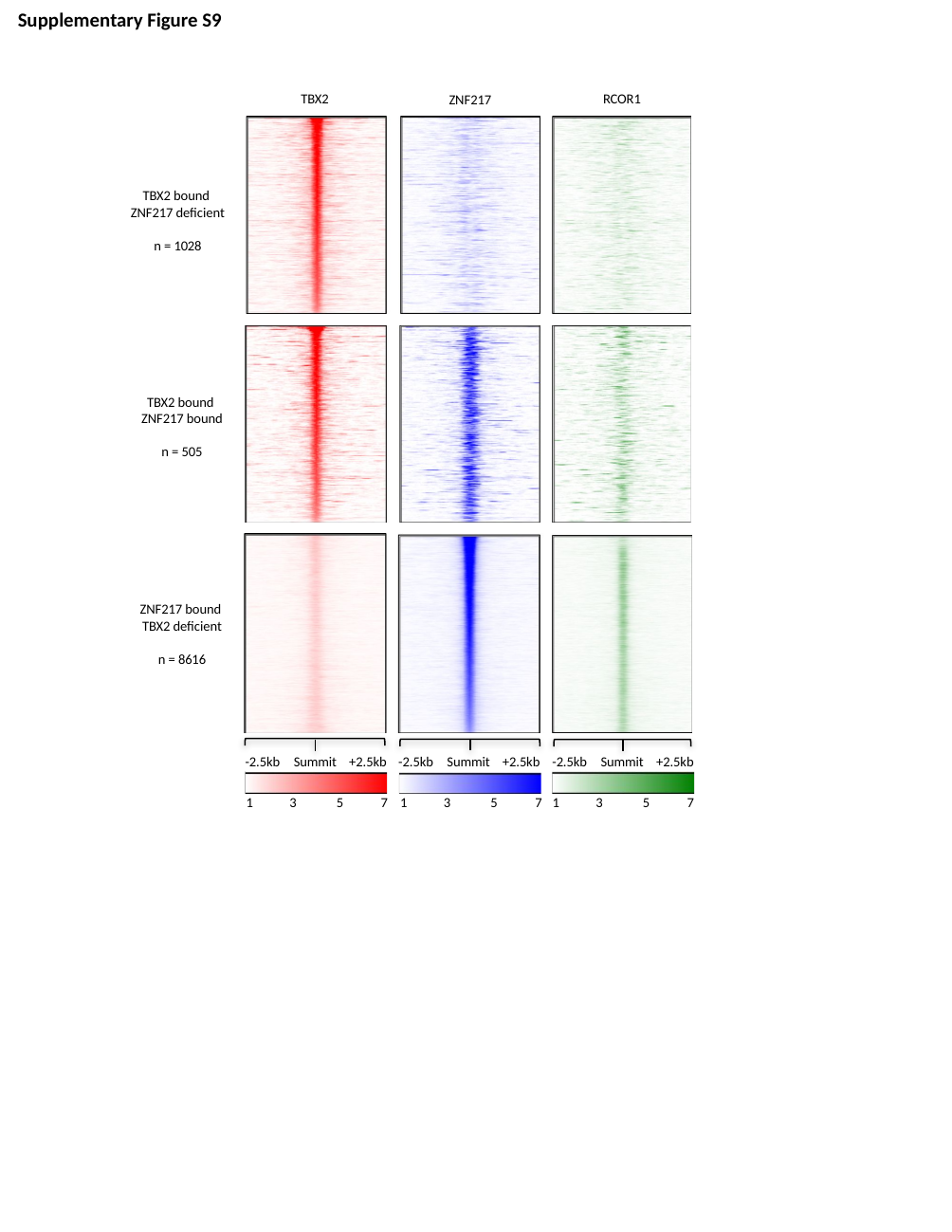

Supplementary Figure S9
RCOR1
TBX2
ZNF217
TBX2 bound
ZNF217 deficient
n = 1028
TBX2 bound
ZNF217 bound
n = 505
ZNF217 bound
TBX2 deficient
n = 8616
-2.5kb
+2.5kb
Summit
-2.5kb
+2.5kb
Summit
-2.5kb
+2.5kb
Summit
1
3
5
7
1
3
5
7
1
3
5
7

## Slide 10
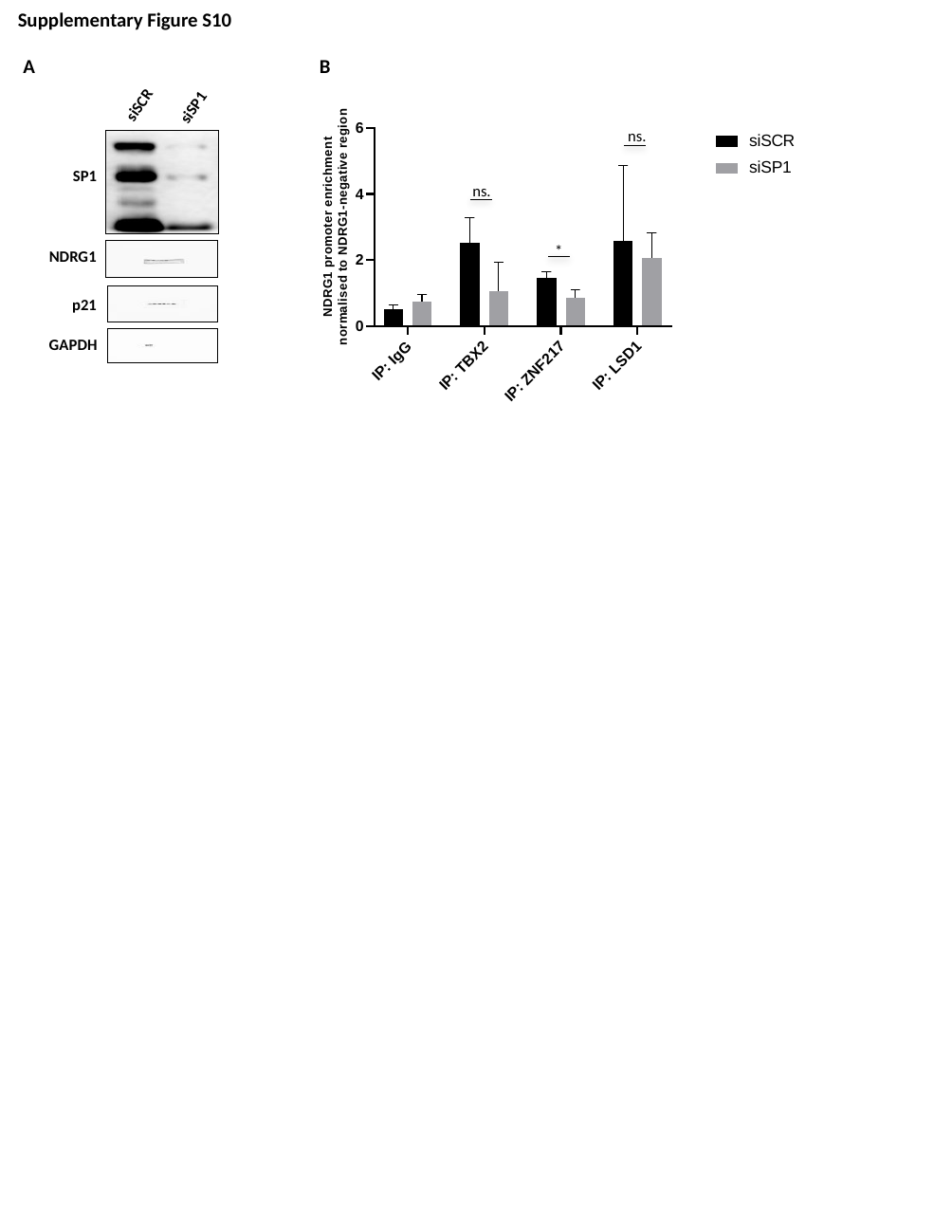

Supplementary Figure S10
B
A
siSP1
siSCR
ns.
SP1
ns.
*
NDRG1
p21
GAPDH

## Slide 11
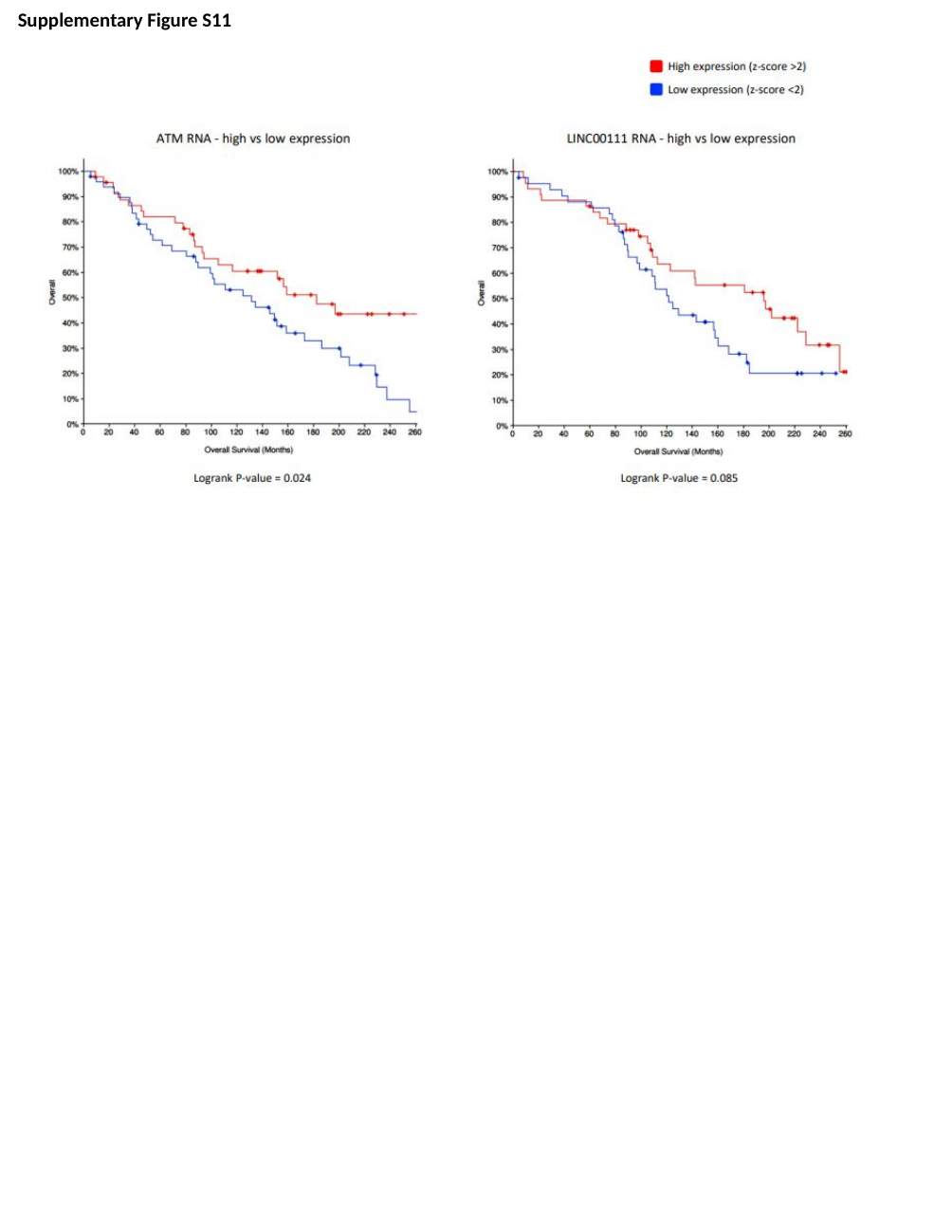

Supplementary Figure S11
Logrank P-value = 0.024
Logrank P-value = 0.085

## Slide 12
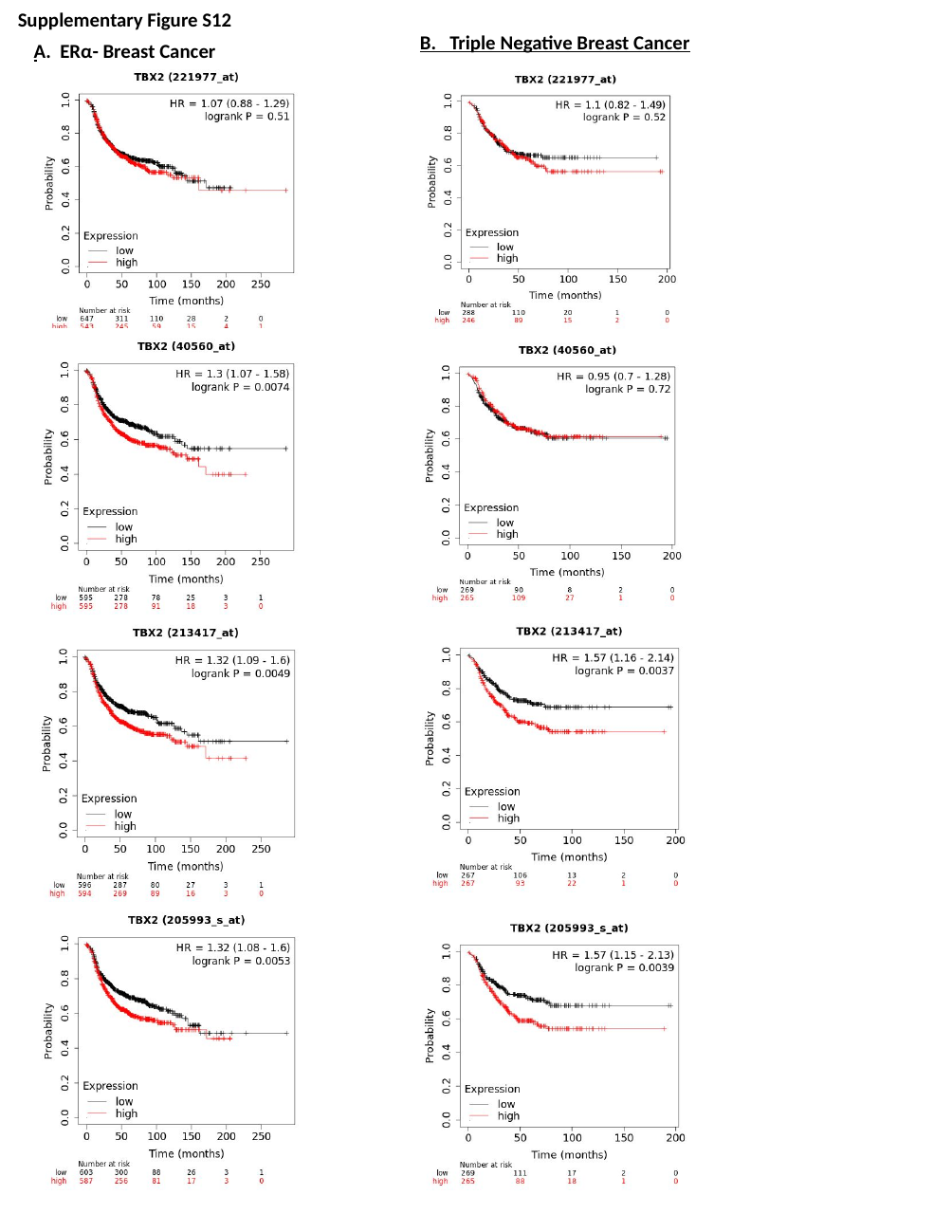

Supplementary Figure S12
B. Triple Negative Breast Cancer
A. ERα- Breast Cancer
