# Supplementary material for: TBX2 acts as a potent transcriptional silencer of tumour suppressor genes through interaction with the CoREST complex to sustain the proliferation of breast cancers
Source: Nucleic Acids Res. 2022 Jun 10;50(11):6154–73. doi: 10.1093/nar/gkac494 (PMC9226508; doi:10.1093/nar/gkac494)
Supplement: gkac494_Supplemental_Files [file gkac494_supplemental_files.zip › McIntyre et al TBX2 NAR Supplementary Material Legends Final.docx]

**Supplementary Figure S1: Extended *in vivo* data for SP-2509 experiment in MCF7 xenografts.** Mice were treated with vehicle or SP-2509 for 4 weeks (as described in Materials and Methods). **(A)** Comparison between vehicle and SP-2509 for absolute tumour volume, and volume relative to day 0 for the same tumour sample. **(B)** Toxicity measurements comparing vehicle and SP-2509 for total body weight, liver weight and kidney weight. Error bars represent mean ± s.e.m. of ten individual mice. ***P*<0.01; ****P*<0.001; ns = not significant.

**Supplementary Figure S2:** **TBX2 co-immunoprecipitation showing SP-2509 treatment disrupts TBX2-ZNF217 interaction in MCF7 cells.** Immunoblots of ZNF217, LSD1, TBX2 and HDAC1 following immunoprecipitation of TBX2 showing interactions with CoREST components, either following treatment with vehicle control (DMSO), or treatment with the LSD1 inhibitor, SP-2509. IgG Light chain expression was used as an immunoprecipitation loading control.

**Supplementary Figure S3:** **TBX2, LSD1 and ZNF217 siRNA knockdowns induce cell cycle alterations in MCF7 cells.** Bar graphs showing % gated cells for sub-G0, G1, S and G2 cell cycle phases in MCF7 cells following treatment with 1μM SP-2509 for 24, 48 and 72 hours. ***P*<0.01; ****P*<0.001; ns = not significant.

**Supplementary Figure S4:** **TBX2, LSD1 and ZNF217 are enriched at the NDRG1 promoter.** Bar graph showing Chromatin-Immunoprecipitation (ChIP) assay qPCR values for pulldowns using TBX2, LSD1 and ZNF217 antibodies, with isotype matched IgG antibody pulldown as negative control. ChIP-PCR values for the NDRG1 promoter were normalised to an upstream region of NDRG1 as a pulldown control. N=3. ***P*<0.01; ****P*<0.001; ns = not significant.

**Supplementary Figure S5: A. TBX2 target gene expression and SP-2509 responses in TBX2 non-expressing cells.** **(A).** Bar graphs showing qPCR values for the TBX2 target genes, NDRG1 and CST6, in two TBX2 non-expressing cell lines (Hs578T and MDA-MB-436) following knockdown of TBX2, LSD1 and ZNF217. **(B).** Images of crystal violet stained clonogenic assays in three TBX2 non-expressing and one TBX2 expressing (MCF7) breast cancer cell lines, following treatment with 250nM SP-2509 or DMSO control. **(C).** Bar graph showing colony number (% relative to DMSO control) for the same three TBX2 non-expressing breast cancer lines and the TBX2-expressing breast cancer line (MCF7), following treatment with the LSD1 inhibitor, SP2509. ***P*<0.01; ****P*<0.001; ns = not significant.

**Supplementary Figure S6: HDAC1/2/3 inhibition by Entinostat upregulates NDRG1 expression. (A)** MCF7 cells were treated with increasing doses of Entinostat (MS-275) for 72h. MTT assay shows viability measurements relative to vehicle control (DMSO); western blot (centre) shows relative levels of histone acetylation, NDRG1 expression and CDK1 expression; time-matched RT-qPCR (right) shows relative expression levels of NDRG1 mRNA. **(B)** T47D cells were treated with increasing doses of Entinostat (MS-275) for 72h and assays conducted as described above (in A). Error bars represent mean ± s.d. GAPDH serves as loading control for all western blots. ***P*<0.01; ****P*<0.001; ns = not significant.

**Supplementary Figure S7: Genome-wide chromatin binding profile of TBX2 in MCF7 breast cancer cells. (A)** TBX2 ChIP-seq conducted in-house on MCF7 cells using ab33298 antibody, compared with input control DNA. Coverage bigWigs were generated by pooling reads from 2 biological experiments; profile plots denote mean read density (counts per million) ± 2.5kb for all TBX2 peak summits consistent between 2 biological replicates. (**B**) Genome browser snapshot comparing TBX2 ChIP with input signal at the most strongly enriched TBX2-bound promoter (*WDR74* gene). Scale bars correspond to reads per million. **(C)** Genome browser snapshot comparing TBX2 ChIP with input signal at the *NDRG1* promoter, centred on location of historic ChIP-PCR primers. Scale bars correspond to reads per million. **(D)** Enrichment q-values from indicated reference databases, with each displaying top 5 terms associated with genes from TBX2-bound promoter regions. **(E)** Position weight matrices (PWMs) of significantly enriched TF motifs concentrated within 100bp of TBX2 peak summits, as identified by STREME. Below pie charts show distribution between promoter and non-promoter regions of all TBX2 binding sites containing the motif.

**Supplementary Figure S8. Genome-wide chromatin binding of TBX2 is cancer tissue-specific. (A)** TBX2 ChIP-seq conducted in-house on MCF7 cells, compared with public ChIP-seq for TBX2 in Kelly cells (GSM2915911). Profile plots denote mean enrichment of ChIP read density over input control read density ± 2.5kb for identified peak summits. 3 analysis groups of regions were characterised corresponding to MCF7 sites lacking significant Kelly signal, MCF7/Kelly overlapping sites, and Kelly sites lacking significant MCF7 signal. **(B)** Distribution of ChIP binding sites between promoter and non-promoter regions corresponding to above analysis group. **(C)** Enrichment q-values for top 5 upstream GEO kinase terms associated with genes from bound promoter regions, corresponding to above analysis group. **(D)** Position weight matrices (PWMs) of significantly enriched TF motifs concentrated within 100bp of ChIP peak summits as identified by STREME, corresponding to above analysis group.

**Supplementary Figure S9. ZNF217 is required for RCOR1 binding at TBX2 sites in MCF7 cells.** Normalised bigWigs were generated for TBX2, ZNF217 and RCOR1 ChIP-seq as fold enrichment of ChIP read density over input read density. Heatmaps display signal enrichment ± 2.5kb for identified peak summits, contiguous with below colour scale. 3 horizontal groups are displayed corresponding to TBX2 peaks lacking significant ZNF217 signal, TBX2/ZNF217 overlapping peaks, and ZNF217 peaks lacking significant TBX2 signal. All TBX2-bound heatmaps are centred on the TBX2 summit and ranked by the TBX2 signal therein; TBX2-deficient heatmaps are centred on the ZNF217 summit and ranked by the ZNF217 signal therein.

**Supplementary Figure S10: Sp1 knockdown impairs TBX2, LSD1 and ZNF217 recruitment to the NDRG1 promoter. (A)** Immunoblots showing efficacy of knockdown of Sp1 siRNA (siSP1), relative to scrambled control (siSCR) siRNA in MCF7 cells. Blots were reprobed with NDRG1 and P21^WAF1^ (p21). Chromatin was prepared from matched knockdowns for the ChIP assay shown in B. **(B)** Bar graph showing Chromatin-Immunoprecipitation (ChIP) assay qPCR values for pulldowns using TBX2, LSD1 and ZNF217 antibodies, with isotype matched IgG antibody pulldown as negative control. ChIP-PCR values for the NDRG1 promoter were normalised to an upstream region of NDRG1 as a pulldown control. N=3. ***P*<0.01; ****P*<0.001; ns = not significant.

**Supplementary Figure S11. Impact of TBX2-CoREST target gene LINC00111 expression on long-term survival of breast cancer patients.** Kaplan-Meier curves demonstrate impact of RNA expression of LINC00111 and ATM (tumour suppressor positive control) on overall survival of pan-breast cancer patients, as determined by analysis of the METABRIC study using the cBioPortal resource (http://www.cbioportal.org/study/summary?id=brca_metabric). High and low RNA-expressing patients are classified as having microarray z-scores for the indicated gene of >2 and <2, respectively. Difference between groups is calculated by logrank test whereby *P*<0.05 is considered statistically significant.

**Supplementary Figure S12: TBX2 expression correlates with poor outcome breast cancers.** Kaplan-Meier plots generated from the online meta-analysis tool, *KM Plotter*, showing the correlation of 4 different TBX2 probe sets with clinical outcome in Estrogen Receptor-α negative (ERα-) and Triple Negative Breast Cancers.
